# Hidden proteome of synaptic vesicles in the mammalian brain

**DOI:** 10.1073/pnas.2011870117

**Published:** 2020-12-21

**Authors:** Zacharie Taoufiq, Momchil Ninov, Alejandro Villar-Briones, Han-Ying Wang, Toshio Sasaki, Michael C. Roy, Francois Beauchain, Yasunori Mori, Tomofumi Yoshida, Shigeo Takamori, Reinhard Jahn, Tomoyuki Takahashi

**Affiliations:** ^a^Cellular and Molecular Synaptic Function Unit, Okinawa Institute of Science and Technology Graduate University, Okinawa 904-0495, Japan;; ^b^Department of Neurobiology, Max Planck Institute for Biophysical Chemistry, D-37077 Göttingen, Germany;; ^c^Instrumental Analysis Section, Okinawa Institute of Science and Technology Graduate University, Okinawa 904-0495, Japan;; ^d^Imaging Section, Okinawa Institute of Science and Technology Graduate University, Okinawa 904-0495, Japan;; ^e^Laboratory of Neural Membrane Biology, Graduate School of Brain Science, Doshisha University, 610-0394 Kyoto, Japan

**Keywords:** synapse, deep proteomics, synaptic vesicles, brain disorders, neurotransmission

## Abstract

Current proteomic studies clarified canonical synaptic proteins that are common to many types of synapses. However, proteins of diversified functions in a subset of synapses are largely hidden because of their low abundance or structural similarities to abundant proteins. To overcome this limitation, we have developed an “ultra-definition” (UD) subcellular proteomic workflow. Using purified synaptic vesicle (SV) fraction from rat brain, we identified 1,466 proteins, three times more than reported previously. This refined proteome includes all canonical SV proteins, as well as numerous proteins of low abundance, many of which were hitherto undetected. Comparison of UD quantifications between SV and synaptosomal fractions has enabled us to distinguish SV-resident proteins from potential SV-visitor proteins. We found 134 SV residents, of which 86 are present in an average copy number per SV of less than one, including vesicular transporters of nonubiquitous neurotransmitters in the brain. We provide a fully annotated resource of all categorized SV-resident and potential SV-visitor proteins, which can be utilized to drive novel functional studies, as we characterized here Aak1 as a regulator of synaptic transmission. Moreover, proteins in the SV fraction are associated with more than 200 distinct brain diseases. Remarkably, a majority of these proteins was found in the low-abundance proteome range, highlighting its pathological significance. Our deep SV proteome will provide a fundamental resource for a variety of future investigations on the function of synapses in health and disease.

## Significance

Mammalian central synapses of diverse functions contribute to highly complex brain organization, but the molecular basis of synaptic diversity remains open. This is because current synapse proteomics are restricted to the “average” composition of abundant synaptic proteins. Here, we demonstrate a subcellular proteomic workflow that can identify and quantify the deep proteome of synaptic vesicles, including previously missing proteins present in a small percentage of central synapses. This synaptic vesicle proteome revealed many proteins of physiological and pathological relevance, particularly in the low-abundance range, thus providing a resource for future investigations on diversified synaptic functions and neuronal dysfunctions.

The functions of eukaryotic cells, in all their complexity, depend upon highly specific compartmentalization into subcellular domains, including organelles. These compartments represent functional units characterized by specific supramolecular protein complexes. A major goal of modern biology is to establish an exhaustive, quantitative inventory of the protein components of each intracellular compartment. Such inventories are points of departure, not only for functional understanding and reconstruction of biological systems, but also for a multitude of investigations, such as evolutionary diversification and derivation of general principles of biological regulation and homeostasis.

Essential to communication within the nervous system, chemical synapses constitute highly specific compartments that are connected by axons to frequently distant neuronal cell bodies. Common to all chemical synapses are protein machineries that orchestrate exocytosis of synaptic vesicles (SVs) filled with neurotransmitters in response to presynaptic action potentials (APs), resulting in activation of postsynaptic receptors. Moreover, synapses are composed of structurally and functionally distinct subcompartments, such as free and docked SVs, endosomes, active zones (AZs) at the presynaptic side, and receptor-containing membranes with associated scaffold proteins on the postsynaptic side. Thus, it is not surprising that mass spectrometry (MS)-based proteomics, combined with subcellular fractionation, yields protein inventories of high complexity. For instance, >2,000 protein species were identified in synaptosomes (1), ∼400 in the SV fraction (2), ∼1,500 in postsynaptic densities (3), and ∼100 in an AZ-enriched preparation (4).

While these studies provide insights into the protein composition of synaptic structures, they are still inherently limited for two reasons. First, synapses are functionally diverse with respect to the chemical nature of their neurotransmitters, as well as their synaptic strength, kinetics, and plasticity properties (5). Therefore, analyzed subcellular fractions represent “averages” of a great diversity of synapses (6) or SVs (2). The second limitation is that proteins known to be present in specific subsets were not found in these studies, despite the unprecedented sensitivity of modern mass spectrometers. In fact, many functionally critical synaptic proteins have remained undetected. For example, the synaptotagmin (Syt) family, major Ca^2+^ sensors of SV exocytosis, comprises >15 members, of which only 5 had been identified in previous SV proteomics (2, 4, 7). Missing isoforms included Syt7, involved in asynchronous transmitter release (8), synaptic plasticity (9), and SV recycling (10). Likewise, the vesicular transporters for monoamines (VMATs) and acetylcholine (VAChT) neurotransmitters were missing in these studies. Clearly, known components of the diversified synaptic proteome have been missing, and it is not possible to predict how many more such proteins remain hidden.

What are the reasons for the continuing incompleteness of the synaptic protein inventory? Proteome identification and quantification rely heavily on MS detectability of peptides generated by digestion of extracted proteins with sequence-specific enzymes, such as trypsin. However, in MS analysis of complex biological samples, peptide signals from a few abundant proteins often mask those that are less abundant. Additionally, the probability of obtaining peptides with similar masses, but different amino acid sequences, increases with increasing sample complexity (11, 12). To overcome these limitations, we have elaborated a workflow with dual-enzymatic protein digestion in sequence combined with an extensive peptide separation prior to MS analysis. As proof of concept, we have utilized purified SV fractions from rat whole brain, which serve as a benchmark for quantitative organellar proteomics (2). As a result, we detected ∼1,500 proteins in the SV fraction, three times more than reported previously. This proteome not only covers all known canonical SV proteins but also contains proteins previously overlooked, such as the low-abundance Syts and SV transporters. Moreover, peptide quantification allowed for differentiating “SV-resident” from “SV-visitor” proteins. In fact, most “SV-resident” proteins revealed in our SV proteomics are of low abundance, with an average copy number of less than 1 per SV, suggesting a larger molecular and functional diversity of SVs than previously thought. Remarkably, more than 200 proteins detected in the SV fraction are genetically associated with brain disorders, 76% of which were previously hidden.

## Results

### A Workflow with Enhanced Peptide Recovery and Separation Greatly Extended Synaptic Proteome Coverage.

**Fig. 1.**
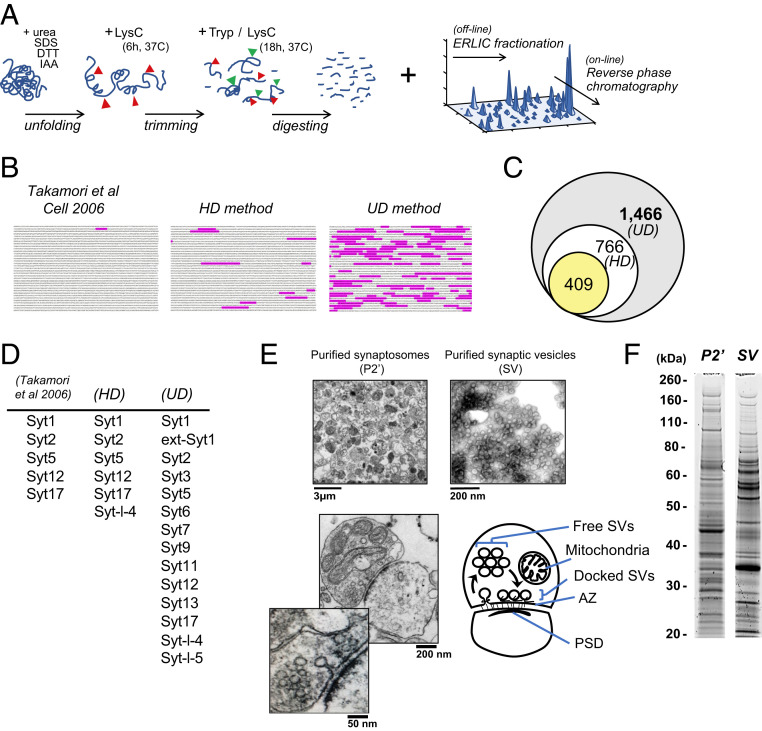
UD proteomics tripled the known SV proteome size. (*A*) Key steps in the UD proteomics method: sequential enzymatic digestion steps followed by orthogonal peptide separations using multiple biophysical properties of amino acids (*SI Appendix*, Fig. S1) (IAA, iodoacetamide). (*B*) Unique peptide coverage by MS of the AZ protein Piccolo (highlighted amino acid sequence) in Takamori et al. (2), HD and UD proteomic methods. (*C*) Numbers of proteins identified in the SV fraction by Takamori et al. (2) (yellow), HD (white), and UD (gray) methods. (*D*) Syt family members identified in the SV fraction by Takamori et al. (2), HD and UD methods. (*E*) EM images of the purified synaptosome (P2′) and SV fractions, showing clear vesicles with diameters of ∼40 nm. (*Bottom*) Representative synaptic structures in the P2′ fraction in EM images, showing intact subsynaptic compartments, as illustrated (PSD, postsynaptic density). (*F*) SDS/PAGE profiles of proteins extracted from the P2′ and SV fractions.

A workflow was developed to increase coverage of protein-specific sequences or “unique peptides” prior to MS identification. First, to increase the number of accessible cleavage sites, we introduced Lys-C treatments before and during tryptic digestion (Fig. 1*A* and *SI Appendix*, Fig. S1). Second, to improve separation of the peptides, we introduced off-line fractionation using electrostatic repulsion-hydrophilic interaction chromatography (ERLIC), based on their charges, polarities, isoelectric pH, posttranslational modifications, and orientations (13, 14), prior to conventional hydrophobicity-based reverse-phase chromatography (RPC). To evaluate the contribution of this workflow to greater protein coverage, we also ran a conventional protein digestion-peptide separation protocol combined with modern mass spectrometer (Q-Exactive Plus) analyses, which we designated as the “high-definition” (HD) method, whereas we refer to our workflow as the “ultra-definition” (UD) method (Fig. 1*B* and *SI Appendix*, Fig. S1). The UD-based proteomics revealed 1,466 proteins in the SV fraction (Fig. 1*C*). This is twice as many as with the HD method (766), and more than three times as many as previously reported (2). The increased sensitivity of the UD method is also evident from the recovery of unique peptides of individual proteins. For instance, 116 unique peptides were identified for the large AZ protein Piccolo whereas the HD method recovered only 14, and only 1 was identified in the previous study (2) (Fig. 1*B*). The UD method increased not only the size of the SV proteome, but also the number of isoforms identified within individual protein families, such as the Syts (Fig. 1*D*), for which most known family members were detected (13 of 15 and extended-Syt1). The previously undetected isoforms include Syt7, which was recently found to regulate multiple modes of neurotransmitter release (8–10). In contrast, the HD method added only one Syt isoform to the previous SV proteome (2).

As expected, the UD method also detected a much greater number of proteins (4,439) in synaptosomal fractions (P2′) than the HD method (1,790) (*SI Appendix*, Fig. S1*A*), indicating that the resolving power of the UD method is based upon improved workflow prior to MS analysis (*SI Appendix*, Fig. S1). Note that each sample used for our MS analyses was checked by electron microscopy (EM) and electrophoresis. Typical synaptosomal profiles were observed in P2′ samples whereas uniform vesicle structures of 40 to 50 nm in diameter predominated SV fractions (Fig. 1*E*). Proteins extracted from the P2′ and SV fractions showed distinct sodium dodecyl sulfate polyacrylamide gel electrophoresis (SDS/PAGE) profiles (Fig. 1*F*).

### Improved Quantification Revealed the Synaptic Organization and Diversity of the SV Proteome.

In quantitative MS, protein abundance can be determined using intensity-based absolute quantification (iBAQ), a label-free approach in which the summed intensities of all unique peptides of a protein are divided by the total number of unique peptides detected. Thus, the increased peptide recovery achieved with the UD method is expected to improve the accuracy of protein quantification. To test this assumption, we performed immunoblot analyses for 41 proteins in the fractions during SV purification and compared with the quantification profiles of the HD and UD methods (*SI Appendix*, Fig. S2). As expected, proteins located at the postsynaptic side or in the synaptic cleft were found in the P2′ fraction, but not in the SV fraction, both in immunoblot and MS analyses (*SI Appendix*, Fig. S2*A*). Proteins residing on SVs were found at higher levels in the SV than the P2′ fraction, in both Western blot and UD analyses (*SI Appendix*, Fig. S2*B*). In contrast, the HD method failed to detect some SV proteins in P2′. Similar inconsistencies between HD iBAQ data and immunoblot profiles were found for proteins in AZ, presynaptic membrane, and cytoplasm (*SI Appendix*, Fig. S2 *C*–*E*): altogether in 30% (13 of 41) of cases. These results highlight the importance of the UD workflow for quantitative proteomics.

**Fig. 2.**
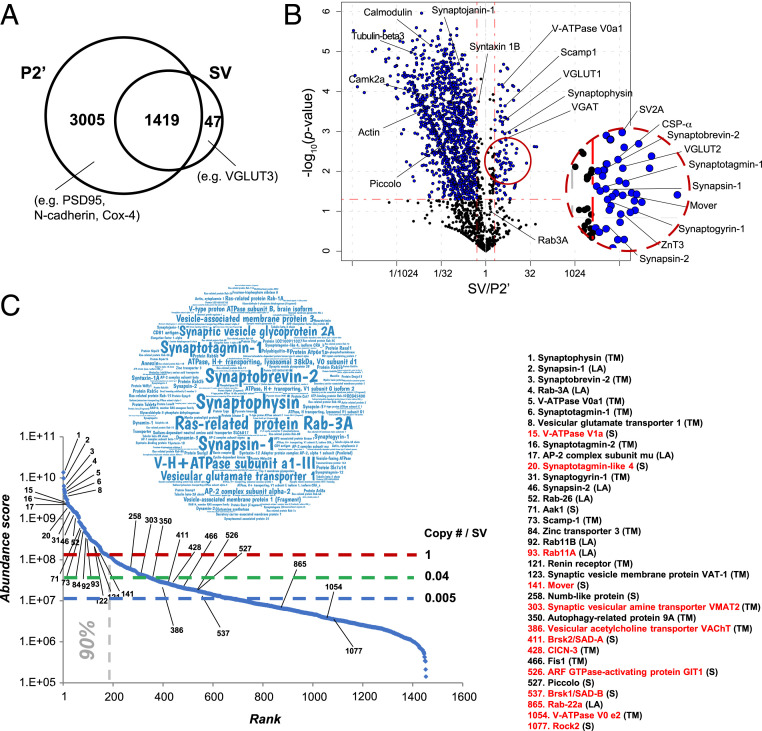
Synaptosomal organization and diversity of the SV proteome revealed by UD proteomics . (*A*) Numbers of synaptosomal proteins detected in either or both P2′ and SV fractions. (*B*) Volcano plot showing synaptosomal protein distribution in SV over P2′ fractions; *x* axis (log_2_ scale), mean iBAQ (SV/P2′) ratio; *y* axis (log_10_ scale), probability of statistical significance (*P* value, from three independent experiments). The horizontal red dashed line indicates *P* = 0.05; vertical dashed lines indicate ratios of 1/2 and 2, respectively. (*C*) Relative abundance of SV proteins in a “word cloud” representation (*Inset*) and in a ranked (iBAQ) abundance curve of the SV proteome. Shown are estimated copy numbers per SV (*SI Appendix*, Fig. S3) are indicated with horizontal dashed lines. The vertical dashed line indicates proteins accounting for 90% of the total mass of SV. Names of representative proteins are indicated. Previously undetected SV proteins are marked in red. Abbreviations in parentheses denote the following: TM*,* transmembrane; LA*,* lipid-anchored; and S*,* soluble proteins.

SVs are purified from synaptosomes (P2′), which contain all SV proteins, whereas SVs may not contain proteins from other synaptic compartments. Of 4,424 proteins in the P2′ fraction, 3,005 were detected only in P2′, including postsynaptic and mitochondrial proteins (Fig. 2*A*). Of 1,466 SV proteins, 1,419 were detected in P2′. The remaining 47 SV proteins were of low abundance, including VGLUT3, a vesicular glutamate transporter isoform present in a limited set of central nervous system (CNS) synapses. To evaluate possible contamination of postsynaptic proteins into the SV fraction, we have referred to the Synaptic Gene Ontologies (SynGO) resource (15). Of all SV fraction proteins, 97 (7%) are annotated as postsynaptic proteins, but 47 out of 97 proteins are reportedly present and function in presynaptic compartments (Dataset S1). Thus, contamination of postsynaptic proteins in the SV proteome seems minor within the proteins detected in the SynGO database.

To distinguish SV residents from proteins transiently interacting (“visitors”) with SVs, we determined the iBAQ ratio SV/P2′ in a volcano plot (Fig. 2*B*). Of 1,466 SV proteins, 134 had an SV/P2′ ratio significantly higher than 2 (*P* < 0.05). We used this criterion to define the bona fide “SV-resident” protein group. It comprised all previously established SV proteins (2, 16, 17), as well as hitherto uncharacterized proteins (see *The “Hidden SV Proteome” Uncovered by the UD Proteomic Method*). On the other hand, a majority of the 1,466 proteins had an SV/P2′ ratio lower than 1, suggesting that these occasionally interact with SVs. We defined them as potential SV-visitor proteins. This repertoire contains 1) cytosolic proteins, such as calmodulin, actin, and synaptojanin-1; 2) AZ proteins, such as Piccolo and Bassoon; and 3) plasma membrane proteins, such as syntaxin-1, all of which interact transiently with SVs, for instance, in the SV trafficking pathway (4, 18, 19). Thus, UD proteomics provide quantitative information to distinguish SV-resident and SV-visitor synaptic protein repertoires.

We next ranked the 1,466 proteins detected in the SV fraction by iBAQ abundance (Fig. 2*C* and Dataset S1). We confirmed that previously reported canonical transmembrane proteins and lipid-anchored proteins were highly abundant (see the word cloud chart in Fig. 2*C*). The 180 most abundant protein species accounted for 90% of the total protein mass of SVs, having iBAQs of >1.2 × 10^8^ (1.2E8) (Fig. 2*C*). The iBAQ of the remaining 1,286 proteins ranged from E5 to E8. Previously, copy numbers per SV were estimated for abundant SV proteins to construct an “average SV” model (2, 6). Using isotope-labeled peptides, we extended the copy number estimate to all other detected SV proteins (*SI Appendix*, Fig. S3 and Table S1). As a calibration standard, we utilized the previously determined copy number of Syt1: 15 (2). The copy number estimated by this method for Rab3A was 10.5, which nearly coincided with the copy number of 10 previously determined by immunoblotting (2), confirming the accuracy of this method. These analyses indicated that copy numbers of many SV proteins are below 1, suggesting that they are present only in subpopulations of SVs or only transiently interact with SVs.

### The “Hidden SV Proteome” Uncovered by the UD Proteomic Method.

**Fig. 3.**
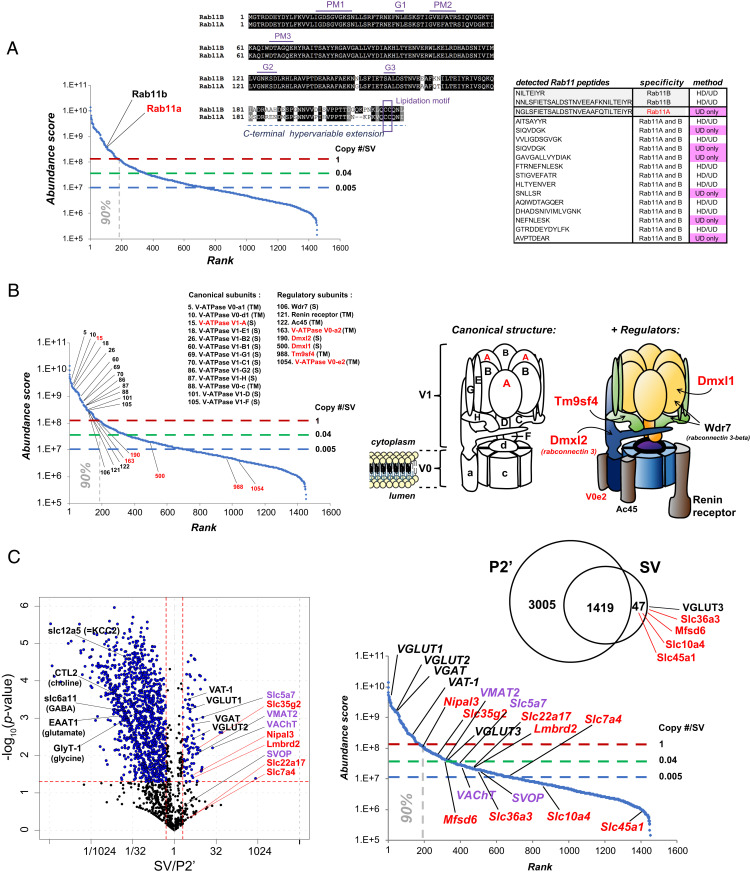
UD proteomics unveiled hidden proteins in both high- and low-abundance ranges of the SV proteome. (*A*) UD proteomics distinguish highly homologous protein isoforms, Rab11A and Rab11B. (*Left*) Positions of Rab11A and Rab11B in the ranked (iBAQ) abundance curve. (*Middle*) Amino acid sequence alignment of Rab11A and Rab11B, showing 91% identity. (*Right*) List of unique Rab11 peptides detected in the SV fraction by HD and UD methods. Rab11A is identified only by UD from a unique peptide at the C-terminal region of Rab GTPase. (*B*, *Left*) V-ATPase–related proteins detected in the SV fraction on the ranked (iBAQ) abundance curve. Right illustration: Structural model of the V-ATPase protein V_0_ (a, c, and d) and V1 (A to H) subunits in SVs. Proteins revealed by UD proteomics are indicated in red. (*C*) SV-resident transporter proteins revealed in the SV fraction by UD proteomics (red), known but missing in previous proteomic studies (purple) in the SV-P2′ volcano plot (*Left*) and Venn diagram (*Right Top*). (*Right Bottom*) Position of the transporters in the ranked (iBAQ) abundance curve of the SV proteome.

To reveal the hidden SV proteome, we tabulated an SV protein inventory detected by UD proteomics with annotations (Dataset S1), including comparisons with those by Takamori et al. (2). This inventory allows one to extract novel insights into SV structure and function using various filters, such as gene family names, abundance rank, and molecular, structural, or functional categories. The first example selected from the inventory is the Rab GTPases, which function in vesicle transport to specific subcellular organelles and membranes (20). They are evolutionally conserved, displaying 75 to 95% amino acid sequence identity. Such high homology has hampered proteomic detection, but, using UD proteomics, we detected and quantified 40 Rabs in the SV fraction, of which 8 were hitherto unreported. Of 32 Rabs previously documented (2), abundance was quantified for only 18 using Western blot analysis (21). We found a majority of high-abundance Rabs (25 of 40) significantly enriched in the SV fraction (*SI Appendix*, Fig. S4*B*). Among them, Rab11A and Rab11B are highly homologous, with 91% amino acid sequence identity (Fig. 3*A*). Despite such similarity, they reportedly function in opposing endosomal sorting routes (22). We found 14 unique peptides common to both Rab11A and -B; however, only UD proteomics could detect a Rab11A signature in the C-terminal hypervariable region. Thus, UD proteomics can reveal highly homologous, but functionally distinct, proteins.

The second example is the vacuolar-type H^+^-ATPase (V-ATPase) protein complex, which operates as an ATP-driven proton pump to energize SVs for neurotransmitter uptake. The V-ATPase complex is composed of a cytoplasmic domain “V1” comprising eight subunits (A to H), and a transmembrane domain “V_0_” assembled from four subunits (a, c, d, and e) (23) (Fig. 3*B*). Previous proteomic studies estimated the copy number of V-ATPase as ∼1 to 2 per SV, but the complete set of V-ATPase proteins remains unidentified (2, 4, 17). Intriguingly, using UD proteomics, we identified all components of the V-ATPase complex, most of which were found in the high-abundance range of the SV proteome (Fig. 3*B* and Dataset S1). Furthermore, V-ATPase accessory proteins Wdr7 and renin receptor (atp6ap2), and previously hidden Dmxl1 and Dmxl2, were all identified (24) (Fig. 3*B*). These low-abundance accessory proteins, in which only renin receptors are categorized as SV-resident (Dataset S1), may regulate V-ATPase complex functions in a restricted subset of SVs. Thus, the UD method can reveal full sets of subunits comprising large protein complexes.

The third example is SV-resident transporter proteins. Solute carrier (“slc”) transporters are transmembrane proteins that control movements of soluble molecules across cellular membranes. To date, more than 400 slc genes have been identified in mammals, of which ∼40% remain uncharacterized with respect to their expression profiles and functions (25). Our UD analysis detected slc transporters both in SV-resident and SV-visitor repertoires (Fig. 3*C*). The latter may include transporters partially internalized from the plasma membrane into SVs during endocytosis (*SI Appendix*, Fig. S5). SV-resident transporters include VGLUT1 (slc17a7) and VGLUT2 (slc17a6) responsible for glutamate uptake, and VGAT (slc32a1) for GABA and glycine uptake, all of which define the molecular identities of the major SV populations in the brain (26), and which occur at high abundance in the SV proteome (Fig. 3*C*). UD proteomics also detected lower abundance SV-resident transporters that were missing in previous SV proteomic studies. These include VMAT2 (slc18a2) (27), ChT1 (slc5a7) (28), VAChT (29), involved in uptake of monoamines or ACh into SV subpopulations, and SVOP of unknown substrate (atypical slc subfamily) (30). In addition to these well-known transporters, UD analyses revealed nine SV-resident transporters (Fig. 3*C*), among which slc10a4 reportedly transports bile acids into SVs to modulate dopamine activity (31). The remaining eight transporters are orphan slcs of unknown function (*SI Appendix*, Table S2). Thus, UD proteomics have unveiled and quantified hidden transporter proteins of both high and low abundance in the SV proteome, having ubiquitous or restricted presence in SV populations.

**Fig. 4.**
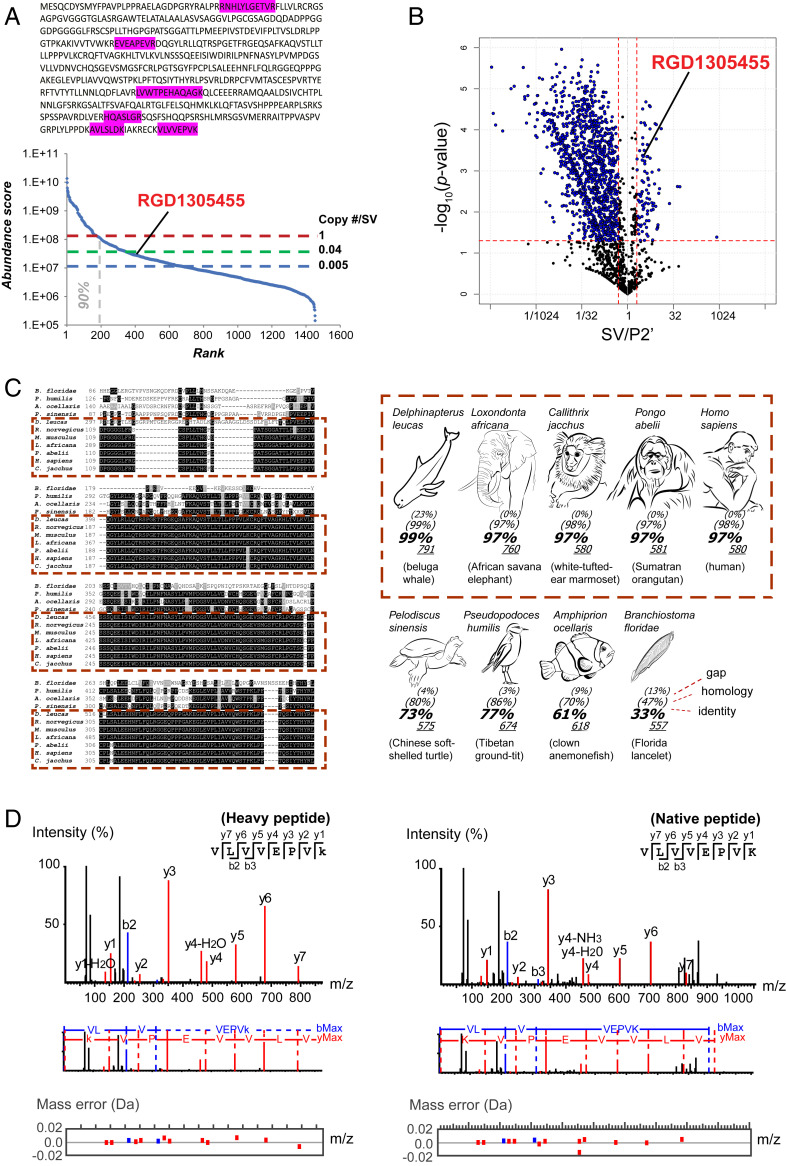
A previously hidden SV-resident protein shows high amino acid sequence homology among mammals. (*A*) “Uncharacterized Protein RGD1305455” (Uniprot ID A0A0G2KAX2), identified in UD proteomics from unique peptides (highlighted within amino acid sequence) and its position in the ranked (iBAQ) abundance plot (*Lower*). No unique peptide could be detected with the HD method. (*B*) SV-resident position of RGD1305455 in the SV-P2′ volcano plot. (*C*) Amino acid sequence comparison of RGD1305455 homologs in various animal species. Black and gray shading indicates identical and similar amino acids, respectively. Dashes represent gaps in sequences. See *SI Appendix*, Table S4 for protein accession numbers and reference sequences. Mammal species are framed in dashed red line boxes (*Left*) and species pictures with >97% identity (*Right*). (*D*) Confirmation of the existence of RGD1305455 protein in the SV fraction using a targeted proteomic approach. VLVVEPVK peptide (detected in UD proteomics) was synthesized using C-terminal “heavier” [^13^C_6_^15^N_2_] lysine (+ eight neutrons = a predefined shift of 8 Da) and was used to track native peptides after mixing with digested SV proteins. (*Left*) MS2 spectra of heavy VLVVEPVK peptide. (*Right*) MS2 spectra of a native peptide detected in the SV fraction that coincides with that of the heavy peptide. Red, blue, and black peaks indicate matched y-ion series, b-ion series, and unmatched ions respectively (*Upper*). (*Middle*) Amino acid sequences corresponding to the ion fragments. (*Lower*) Plotted mass errors of detected versus expected peptide fragments. Errors of native peptide fragments were all <0.02 dalton. Expected masses of all fragments are specified in *SI Appendix*, Table S5.

The fourth example is a discovered protein in the SV fraction (Fig. 4). In data banks, this protein is known as RGD1305455 (Uniprot ID A0A0G2KAX2) or as “uncharacterized protein C7orf43 homolog” and “similar-to-hypothetical protein FLJ10925.” Nothing is known regarding its tissue expression, developmental profile, or subcellular localization. Six unique peptides from RGD1305455 were detected only in UD experiments (Fig. 4*A*). RGD1305455 was found as an SV-resident protein (SV/P2′ ratio = 3) of low abundance (rank 407; copy number/SV ∼0.04) (Fig. 4 *A* and *B*). It harbors a conserved DUF domain (DUF4707) and lacks a predicted transmembrane domain. Database searches revealed that the protein is highly conserved among mammals (>97% amino acid identity) (Fig. 4*C* and *SI Appendix*, Table S3). To confirm its presence in the SV fraction, we employed a targeted proteomic strategy. The UD unique peptide VLVVEPVK (Fig. 4*A*) was chemically synthesized using a “heavy” C-terminal lysine (^13^C_6_ and ^15^N_2_) and mixed with a digested SV protein sample. A parallel reaction monitoring (PRM) assay based on elution time, ionization, and fragmentation of the heavy peptide detected a matched VLVVEPVK peptide in the SV sample (Fig. 4*D*). Close comparison between observed and expected peptide fragments (*SI Appendix*, Table S4) indicated that mass errors of native fragments fell within 0.02 dalton (Fig. 4*D*), confirming with high precision that protein RGD1305455 indeed exists in the SV fraction. Likewise, in PRM assays using 15 other heavy peptides for hitherto unidentified SV-resident proteins (*SI Appendix*, Table S5), the presence of all of the tested proteins in the SV fraction was confirmed.

### Functional Characterization of an SV-Associated Kinase Protein, Aak1.

**Fig. 5.**
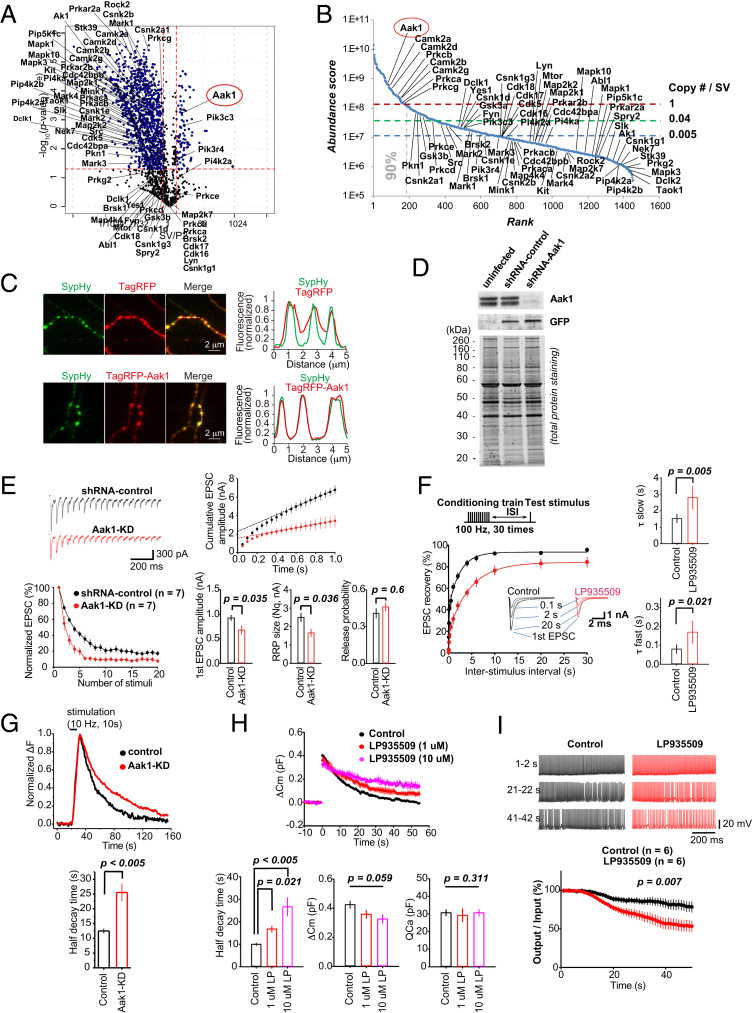
Aak1 is an SV-attached protein kinase essential for high-frequency neurotransmission. (*A*) SV/P2′ volcano plot of 69 kinases detected in the SV fraction by UD proteomics. Labels indicate names of genes encoding proteins. Aak1 (circled in red) is one of the few identified SV-residents. (*B*) SV kinases in the abundance curve. Aak1 is the most abundant of SV kinases. (*C*) Colocalization of exogenously expressed Aak1 and synaptophysin (SypHy) in cultured hippocampal neurons. (*Upper*) Control TagRFP (red) and SypHy (green) images and their line-scanned profiles (*Right*). (*Lower*) TagRFP-Aak1 (red) and SypHy (green) images and their line-scan profiles showing complete colocalization. (*D*) Lentivirus KD efficiency of Aak1 confirmed at DIV15 by Western blot analysis. Hippocampal cells were infected at DIV 11 to 12 with lentivirus coexpressing GFP and shRNA targeting Aak1. (*E*) Aak1-KD enhanced STD of EPSCs and reduced estimated readily releasable pool (RRP) size of SVs (*Nq*) evoked by a 20-Hz train of stimulation in Aak1-KD (red, *n* = 7) or shRNA control (black, *n* = 7) neurons. (*F*) LP935509 (1 μM) slowed both fast and slow components of recovery from STD (100 Hz) at the calyx of Held (red traces, *n* = 6, stimulation protocol indicated on the *Top*). EPSCs at different interstimulus intervals (ISIs) are shown in *Insets*. (*G*) Aak1-KD slowed endocytic SV fluorescence in response to stimulation in pHluorin analyses in hippocampal neurons. Aak1-KD (red), *n* = 16 neurons, 51 boutons; and control (black), *n* = 10 neurons, and 20 boutons. (*H*) LP935509 (1 or 10 μM) prolonged the SV endocytic half-time (*P* < 0.001, *n* = 6 at 1 μM and *n* = 4 at 10 μM: 4; one-way ANOVA: *F*_(2,__13)_ = 16.81] without affecting exocytic [*P* = 0.059, *F*_(2,__13)_ = 2.53] or presynaptic Ca^2+^ current magnitudes [*P* = 0.511, *F*_(2,__13)_ = 0.15] in membrane capacitance measurements at the calyx of Held. (*I*) LP935509 (1 μM) impaired fidelity of glutamatergic neurotransmission at 100-Hz at the calyx of Held. (*Top*) Postsynaptic APs evoked by presynaptic APs in the presence (red) or absence (black) of LP935509 in presynaptic terminals. (*Bottom*) Percentages of postsynaptic APs elicited by presynaptic APs, controls (*n* = 6) and the Aak1 inhibitor (*n* = 6).

The SV fraction contained numerous nontransmembrane proteins, some of which reside with SVs within the synaptic compartment (Dataset S1). These proteins might play a regulatory role in neurotransmission. To address this, we focused our analyses on protein kinases, which are mostly soluble cytoplasmic proteins. We identified AP2-associated protein kinase 1 (Aak1) as an abundant and SV-resident kinase (Fig. 5 *A* and *B*). The copy number of Aak1 was calculated as 1.5/SV (*SI Appendix*, Fig. S3 and Table S1), suggesting a ubiquitous presence among SVs in central synapses (Fig. 5*B*). The enriched profile of Aak1 in the purified SV fraction was confirmed by Western blot, contrasting with other cytoplasmic kinases found in P2′, such as MARK2 or TNiK (*SI Appendix*, Figs. S2*E* and S6*B*). In cultured hippocampal neurons, strong colocalization of exogenously expressed Aak1 (TagRFP-Aak1) with an SV marker, synaptophysin-pHluorin (SypHy), was observed (Fig. 5*C*).

We employed both genetic and pharmacological approaches to clarify the functional role of Aak1, using short hairpin RNA (shRNA) knockdown (KD) of Aak1 expression in cultured hippocampal neurons, and by infusing an Aak1-specific inhibitor, LP-935509 (32), directly into the calyx of Held presynaptic terminals in brainstem slices of rats at postnatal day (P) 13 to 15. For Aak1-KD, we applied a lentivirus targeting Aak1 at day 11 in vitro (DIV11), when synaptophysin became detectable in Western blot (*SI Appendix*, Fig. S6*A*). At DIV15, the KD effect became maximal, reducing Aak1 expression below 5% (Fig. 5*D*). In hippocampal culture at DIV15, excitatory postsynaptic currents (EPSCs) in Aak1-KD neurons underwent a rapid short-term depression (STD) during stimulation at 20 Hz. The magnitude of STD was significantly greater than that in controls (*P* < 0.05, *n* = 7) (Fig. 5*E*). Consistently, at the calyx of Held loaded with LP-935509, EPSCs underwent stronger STD during a 100-Hz train compared to controls (0.3 s, *P* < 0.05, *n* = 7) (*SI Appendix*, Fig. S6*C*). Cumulative histograms of EPSC amplitudes provided the pool size of readily releasable SVs and release probability, indicating that both Aak1-KD (Fig. 5*E*) and Aak1 inhibitor (*SI Appendix*, Fig. S6*C*) reduced the pool size without affecting the release probability. Furthermore, the recovery from STD was prolonged, both at the Ca^2+^-dependent fast component (33) and Ca^2+^-independent slow component at the calyx of Held (Fig. 5*F*). These results together suggest that Aak1 normally facilitates SV recycling, thereby maintaining the releasable SV pool.

To further investigate whether Aak1 is involved in exo-endocytosis of SVs, we performed pHluorin assays in cultured hippocampal neurons (Fig. 5*G*) and capacitance measurements at the calyceal terminal (Fig. 5*H*). In pHluorin assays, endocytic fluorescence half-decay time was prolonged by twofold (*P* < 0.005, *n* = 51) compared to controls (*n* = 20). Likewise, in capacitance measurements, LP-935509 (1 or 10 μM) significantly prolonged the endocytic capacitance change. Capacitance measurements did not indicate a significant reduction of exocytosis. Thus, both at hippocampal and brainstem synapses, Aak1 likely plays an accelerating role in SV endocytosis.

Since the above results suggest involvements of Aak1 in the SV recycling pathway, we further investigated whether Aak1 might have a physiological role in the maintenance of neurotransmission. Simultaneous recordings of presynaptic and postsynaptic APs indicated that the Aak1 inhibitor (1 μM) significantly impaired the fidelity of neurotransmission, assayed as a ratio of postsynaptic APs generated in response to presynaptic APs (*P* < 0.01, *n* = 6) (Fig. 5*I*). Altogether, our data indicate that Aak1 is a canonical SV-resident protein with an essential functional role in maintenance of neurotransmission, particularly at high frequency.

### Many Low-Abundance SV Proteins Are Linked to a Diverse Range of Physiological Functions and Neurological Disorders.

**Fig. 6.**
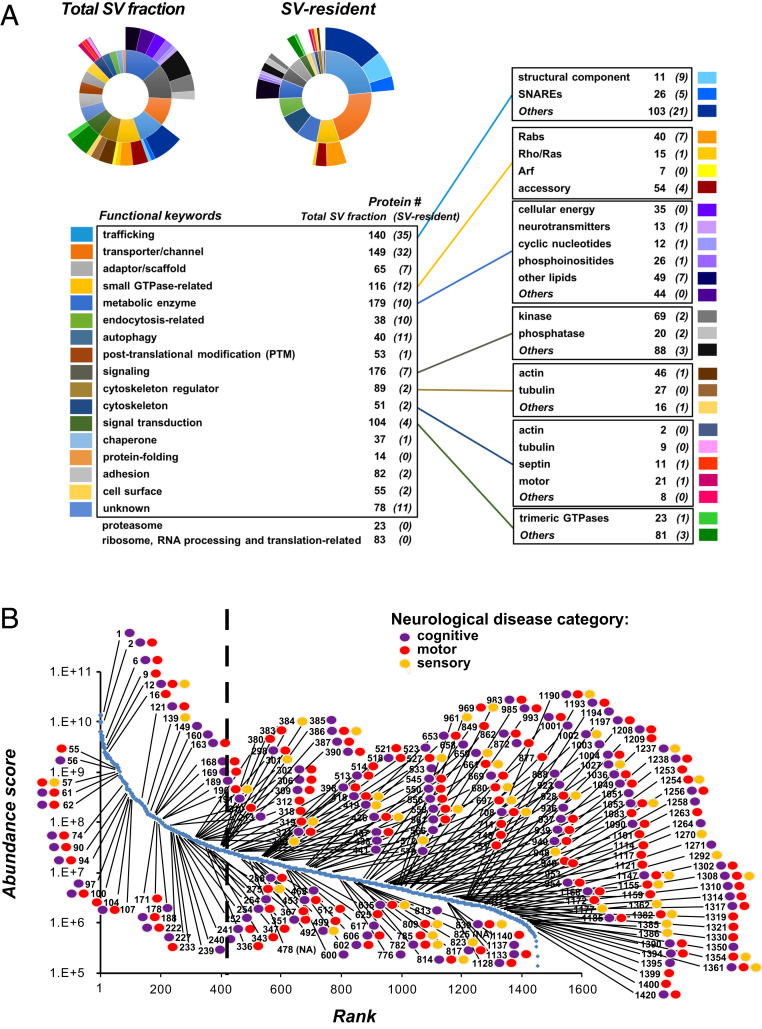
Diversity of neuronal functions and dysfunctions related to the UD-SV proteome. (*A*) Functional mosaic of the SV proteome. Each protein detected in the SV fraction by UD proteomics was associated with one or more functional keywords. (*Top Left*) Sunburst diagrams show distributions of the functional categories (inner circle) and subcategories (outer circle) represented in the total SV fraction proteome and in the SV-resident repertoire. Shown is a list of functional keywords (*Left*) and subcategories (*Right*) with number of proteins representing each category (for details of protein functional annotations, see Dataset S1). (*B*) The deep low-abundant SV proteome is related to brain diseases. Proteins detected in the SV fraction having “disease(s) caused by mutation(s) affecting the gene represented in the entry” were marked, and their rank in the iBAQ-abundance curve is specified. The analysis was performed manually using the Uniprot and GeneCards databases for human diseases. Markers indicate proteins associated with cognitive (purple), motor (red), and/or sensory processing (yellow) disabilities. The vertical dashed line indicates rank 409, the number of proteins identified by a previous SV proteomics study [Takamori et al. (2)]. Proteins to the right hand of the dashed line were mostly revealed by UD proteomics (see Dataset S1 for a listing of all disease names and protein associations).

Many proteins were uncovered by UD proteomics in both high- and low-abundance ranges of the SV fraction proteome, with >80% found in lower ranges (Dataset S1). Even though expressed at low abundance, SV proteins may play important physiological roles. We investigated this possibility using functional and disease annotations in our database, by classifying SV proteins into 17 functional categories with 26 subcategories (Fig. 6*A*). Our dataset contains trafficking proteins including SNAREs involved in various membrane fusions (26 protein species) (2, 6). It also contains many types of Rab GTPases (40 species) and membrane-tethering Trapp complexes (14 proteins) (*SI Appendix*, Fig. S4). Many of these proteins are identified as SV-resident, suggesting that SVs may be equipped with proteins for various trafficking routes toward other presynaptic organelles. Other major categories included proteins involved in signaling (e.g., kinases, phosphatases), signal transduction, and transport of small molecules. UD proteomics detected a high number of metabolic enzymes (179 species), including those involved in neurotransmitter metabolism (13 species), cellular energy production (35 species), lipid regulation (75 species), and cyclic nucleotide second messengers (12 species). These data suggest the occurrence of metabolic reactions on SVs in crowded presynaptic terminals (6). UD proteomics also detected SV proteins categorized as autophagy-related proteins (40 protein species) (*SI Appendix*, Fig. S4*D*). The presence of both SV-resident (e.g., snap29, atg9a, trappc8, and pik3c3) and SV-visitor autophagy-related proteins (e.g., beclin-1, uvrag, map1lc3a, and cisd2) in our SV proteome (*SI Appendix*, Fig. S4*D* and Dataset S1) suggests that autophagic degradation may participate in the maintenance of SV population size within presynaptic terminals.

To examine the pathological implications of our UD proteomics data, we searched for genetic information on SV proteins regarding their associations with neurological diseases (see “*Diseases in the SV fraction*” in Dataset S1) and marked them in ranked abundance plots of SV (Fig. 6*B*) and P2′ fraction proteomes (*SI Appendix*, Fig. S7). We found that 236 different brain diseases are associated with 210 high- and low-abundance proteins of the SV proteome, of which 159 (76%) are revealed by the UD method. Likewise, 55% of these SV proteins were found in low-abundance ranges of the P2′ proteome (*SI Appendix*, Fig. S7). These results indicate the pathological significance of SV proteins irrespective of their abundance. SV protein-associated diseases include many motor (145 associated proteins), cognitive (135 proteins), and sensory system phenotypes, such as visual (33 proteins) and auditory (14 proteins) phenotypes. The database also indicates SV proteins associated with phenocopy diseases, such as mental retardation (28 disease phenotypes), epilepsy (25 phenotypes), Parkinson’s disease (13 phenotypes), amyotrophic lateral sclerosis (4 phenotypes), Alzheimer’s disease (4 phenotypes), and cerebellar ataxia (10 phenotypes). Our UD cross-analyses between functions and diseases indicate that phenocopies may involve proteins from both SV-resident and SV-visitor repertoires, from both high- and low-abundance ranges, and from functionally distinct proteins in the SV life cycle. For example, Parkinson’s disease can be linked to mutations in SV-resident proteins such as renin-receptor (121st rank), involved in SV acidification, dnajc13 (318th rank, SV endocytosis) and sv2c (97th rank, SV trafficking), or in SV-visitor proteins, such as synaptojanin-1 (351st rank, SV endocytosis) or pla2g6 (653rd rank, lipid composition) (Dataset S1). Altogether, our data analyses illustrate the complexity and physiological importance of low-abundance SV protein repertoires revealed by UD proteomics.

## Discussion

In this study, we have used SVs purified from rodent brain as a model for identifying and quantifying the “deep proteome,” applying our proteomic workflow. SVs isolated from mammalian brain are morphologically homogeneous (34) and share a set of common proteins, with more than 90% containing the major SV protein synaptophysin (2). Yet, they are heterogeneous with respect to synapse types and neurotransmitter content. With the proteomic workflow introduced here, we identified ∼1,500 proteins in SVs, more than three times as many as previously reported (2, 4, 7). Of these, we found 134 SV-resident proteins, of which 86 are of low abundance (<1 copy per SV). These proteins may therefore be restricted to SV subsets, deduced from the findings that they include previously missed vesicular transporters for monoamines and acetylcholine, present in only a small percentage of brain synapses. Of the ∼1,500 SV-fraction proteins, more than 200 have genetic associations with CNS diseases, highlighting the importance of this deep diverse and previously hidden proteome for proper brain functions. A resource database (Dataset S1) was constructed to include all data on identification, quantitative distribution, and structural and functional annotations for each protein detected in the SV fraction.

The increased peptide coverage of the “UD workflow” is based on two major improvements in combination: 1) enhanced cleavage using proteases in sequence and 2) the introduction of an off-line orthogonal peptide separation prior to reversed-phase liquid chromatography tandem mass spectrometry (LC-MS/MS). These steps resulted in a remarkable increase in unique peptide detection and have greatly expanded the protein inventory of SVs, including highly homologous proteins within families. For example, 40 Rab proteins, having high sequence homology (75 to 95%), but distinct trafficking functions (20), were identified. Likewise, functionally characterized but hidden Syts, such as Syt7 (8–10), were detected, together with other family members of unknown functions. Moreover, the high peptide yield of UD proteomics allows unprecedented label-free and highly reliable quantification of most proteins in the dataset. We were able to evaluate the copy numbers of many hundreds of proteins, thereby providing a quantitative scope of the whole SV proteome organization. When compared to previous quantitative studies (2, 6), the results largely confirm copy numbers on average per vesicle, except for three proteins—SNAP29, vti1a, and ClC3—that have abundance scores too low to be further considered as major SV proteins. On the other hand, most detected proteins had copy numbers less than 1 per SV on average, revealing much greater SV heterogeneity than previously envisaged.

Our label-free quantification also allowed a quantitative comparison of the proteomes of isolated nerve terminals and purified SVs. This was not only the foundation for identifying bona fide SV residents, but also for distinguishing between SV resident and potential SV visitor proteins. Remarkably, about 50% of the SV residents are nontransmembrane proteins (Dataset S1), highlighting the high degree of proteome organization, despite molecular crowding at the synapse (6). For example, UD proteomics revealed that, among nontransmembrane proteins, Aak1 is a major SV-resident protein, having an SV/P2′ ratio of ∼4 and a copy number/SV of 1.5. Our functional assays indicated that this kinase is essential to maintain high-frequency neurotransmission by accelerating SV recycling. Thus, our classification of SV protein repertoires may facilitate functional studies and may result in the identification of major regulators of synaptic transmission.

It needs to be borne in mind that SVs, starting from enriched synaptosomes, are isolated solely based on their size and density. Therefore, heterogeneity may also be caused, at least in part, by the presence of membranes derived from different trafficking steps, such as partially clathrin-uncoated vesicles, small endosomal vesicles, or SVs from axonal compartments en route to nerve terminals. While these compartments are part of the same recycling pathway and are expected to share vesicular membrane-resident proteins, the “visitor” proteins are likely to be different. This may explain the presence of endosomal-related proteins (e.g., Stx7, AP3) or proteins of the AZ (e.g., Piccolo, Bassoon) in the SV proteome. Moreover, we could not exclude the possibility that the SV preparation is contaminated, even to a small extent, with vesicles from other sources: for instance, small vesicles artificially generated from larger membranes during homogenization, or vesicles from the postsynaptic side. Indeed, analysis of the UD-SV proteome using the SynGO resource (15) has revealed a postsynaptic contamination of at least 4% based on 691proteins that were annotated in SynGO. Regarding possible contaminants in the remaining 775 SV proteins in our study, we cannot make a definitive calculation as these are not annotated in the SynGO database.

A closer look at the defined SV-resident repertoire (proteins with SV/P2′ iBAQ ratios of >2) (Dataset S1) provides important leads toward a better understanding of SV molecular and functional heterogeneity. Of 134 SV-resident proteins, 86 have copy numbers <1/SV (*SI Appendix*, Fig. S8). The 40 most abundant SV-resident proteins (in ranks 1 to 180) include all of the subunits of V-ATPase, vesicular “tetraspanins” including SCAMPs, synaptophysins, and synaptogyrins, Syts and SV2 proteins, as well as membrane-associated synapsins and CSPs. VGLUT1/2 and VGAT, vesicular transporters of the two major neurotransmitters in the brain, glutamate (excitatory synapses) and γ-aminobutyric acid (GABA) (inhibitory synapses), are also in this list. All these proteins are likely present on SVs throughout the entire nervous system.

Minor SV residents (<1 copy per SV, beyond rank 180) include proteins generally involved in membrane trafficking, such as additional SNAREs, Rab GTPases, phospholipid kinases, tethering complexes, and autophagy-related proteins. Their low abundance suggests that they reside on a subset of vesicles within synapses. For example, the copy number of the transmembrane protein Atg9a was 1 per 25 SVs (*SI Appendix*, Fig. S3 and Table S1), implying that 4% of vesicles in the synaptic compartment may be recruited to an autophagic pool. As another possibility, these proteins may be expressed specifically in a small subset of synapses in specific brain regions. Indeed, this list includes the known scarce neurotransmitter vesicular transporters VMAT2, VAChT, Slc5a7, and VGLUT3, reflecting the functional heterogeneity of synapses (Fig. 3*C* and *SI Appendix*, Table S2). Interestingly, our list also includes almost a dozen of hitherto unreported transporter proteins.

Many SV proteins, whether classified as residents or potential visitors, may have specific functions in regulating or maintaining the performance of synapses. In fact, our UD proteomics have detected over 200 proteins in the SV fraction known to be genetically associated with neurological (mental, motor, and sensory processing) disorders. Remarkably, a majority of these proteins (76%) were found in low-abundance ranges and had copy numbers of <0.04/SV. These neurological disorders likely originate from various synaptic dysfunctions specific to discrete neuronal populations of the nervous system. In fact, recent evidence supports the idea of “synaptopathies” as a causal polygenic mechanism for psychiatric diseases (35–38). In the process of evolution, abundant canonical proteins are often ancestral components whereas proteins of low abundance tend to emerge for new functions (39). In this respect, the deep diversified synaptic proteome may account for mammalian- or human-specific neurological diseases. This could be a key reason why, despite technical difficulties, investigations of deep subcellular proteomes beyond “average models” are necessary.

## Materials and Methods

All animal experiments were performed in accordance with guidelines of the Physiological Society of Japan, the German Animal Welfare Act, and regulations at the Okinawa Institute of Science and Technology, at the Max-Planck Institute for Biophysical Chemistry, and at Doshisha University.

### Brain Synaptosomes and SV Purifications.

Synaptosomes (P2′) and SVs were purified from whole brain of 4- to 6-wk-old Sprague–Dawley rats following the same protocols used in ref. 2 and previously described in ref. 34 for SV, and in ref. 4 for P2′. The quality of all P2′ and SV purification procedures was controlled by Western blots of synaptic protein markers and by EM. Extended descriptions of biochemical, imaging, and electrophysiological procedures and analyses are provided in *SI Appendix*, *SI Materials and Methods*.

### UD Proteomics Sample Preparation and MS.

#### Sequential protein digestion.

Fifty micrograms of proteins extracted from P2′ or SV were resuspended into 200 μL of buffer containing 8 M urea, 100 mM Tris⋅HCl, pH 8 (“urea buffer”) and placed onto a Pall Nanosep Omega filter (Sigma). After shaking 1 min at 850 rpm at room temperature (Eppendorf Thermomixer), samples were centrifuged during 13 min at 6,400 × *g* (conditions that were optimal to remove all of the liquid from the upper chamber using a TOMY Kintaro KT-24 centrifuge). After repeating these steps two times, proteins were resuspended with 200 μL of urea buffer containing 50 mM iodoacetamide and incubated in darkness for 1 h at room temperature. The alkylation was then stopped by centrifuging as above and by resuspending protein samples with 200 μL of urea buffer containing 25 mM dithiothreitol. Unfolded proteins were subsequently washed with 20 mM ammonium bicarbonate. Proteolytic enzymes were used in a ratio of 1:50 with proteins. To generate peptides, a first digestion step (“trimming”) was performed using endoproteinase lys-C (Promega) for 6 h at 37 °C, followed by a second digestion step overnight (16 to 18 h) at 37 °C using a trypsin/lys-C combination (Promega). After centrifugation as above, digested peptides were acidified with 1% trifluoroacetic acid, concentrated, and dried using an EZ-2 Elite evaporator (SP Scientific).

#### Orthogonal peptide separations.

To separate peptides, off-line ERLIC or ERLIC-based separation was performed (14). The following conditions were adapted and optimized to obtain the highest number of identified proteins from P2′ and SV samples. Mobile phase solvent preparation was as follows: Solvents were freshly prepared for each experiment using liquid chromatography/mass spectrometry grade acetonitrile (ACN), formic acid (FA), and water from Thermo Fisher Chemicals. Solvent A was prepared as follows: 90% ACN, 0.1% FA. Ammonium hydroxide (NH_4_OH, 25% weight/weight [wt/wt] in water; Fluka) was then added to adjust pH at 4.5. Solvent B was prepared as follows: 30% ACN, 0.1% FA. The digested peptide mixture was resuspended with 20 µL of solvent A and injected into a weak anion exchange PolyWax column (1-mm inner diameter × 150 mm, 5-mm particle size, 300-Å pore size; PolyLC Inc.) using a PAL autosampler (CTC Analytics) for automatic injection and fractions collection, using a gradient mode (3 min solvent A, to 10% B in 7 min, 10% B to 25% B in 24 min, 25% B to 70% B in 16 min, 70% B to 81% B in 6 min, 81% B to 100% B in 3 min, with final wash at 100% B for 6 min and reequilibration at 100% A for 20 min) at a flow rate of 40 μL/min. Twenty-four fractions were collected every 3 min between 0 and 72 min and subsequently concentrated to dryness using a speed vacuum Genevac EZ-2 Elite (SP Scientific).

#### MS.

Dried peptides were resuspended in 30 µL of 0.1% FA and analyzed using a Q-Exactive Plus Orbitrap hybrid mass spectrometer (Thermo Scientific) equipped with an Ultimate 3000 nano-high-pressure liquid chromatography (nano-HPLC) system (Dionex), HTC-PAL autosampler (CTC Analytics), and a nanoelectrospray ion source. Five microliters of each sample were injected into a Zorbax 300SB C18 capillary column (0.3 × 150 mm; Agilent Technologies) and heated at 40 °C. A 1-h HPLC gradient was employed (1% B to 32% B in 45 min, 32% B to 45% B in 15 min, with final wash at 75% B for 5 min and reequilibration at 1% B for 10 min.) using 0.1% FA in distilled water as solvent A, and 0.1% FA in ACN as solvent B. A flow rate of 3.5 μL/min was used for peptide separation. Temperature of the heated capillary was 300 °C, and a 1.9-kV spray voltage was applied to all samples. The mass spectrometer settings were as follow: full MS scan range 350 to 1,500 *m/z* with a mass resolution of 70,000, 30-μs scan time, and automatic gain control set to 1.0E6 ions, and fragmentation MS2 of the 20 most intense ions.

#### Protein identification.

Protein identification was done using Proteome Discoverer software v2.1 (Thermo Scientific) and Mascot 2.6 (Matrix Science) as a search engine. A database downloaded from UniprotKB *Rattus norvegicus* (proteome ID UP000002494) was used with search parameters as follows: trypsin enzyme, up to two miscleavages, with precursor and fragment mass tolerance set to 10 parts per million and 0.02 Da, respectively. Cysteine carbamidomethylation, methionine oxidation, asparagine and glutamine deamidation, and N-terminal protein acetylation were set as variable modifications. The results were filtered using a false discovery rate of <1% as a cutoff threshold, determined by the Percolator algorithm in Proteome Discoverer software.

#### Quantitative proteomic data statistical analyses.

For the volcano plot, iBAQ data obtained from Proteome Discoverer were used for statistical analysis using R software version 3.2.5 (R Project for Statistical Computing). Quasi-Poisson generalized linear models were generated (y ∼1, y ∼ treat) and compared using analysis of deviance for generalized linear model fits (Anova) to obtain *P* values, using an F-test, and adjusted with the Benjamini–Hochberg method.

## Supplementary Material

Supplementary File

Supplementary File

## Data Availability

Proteomic raw data files data have been deposited in the Japan Proteome Standard Repository Database (accession no. JPST000968). All other study data are included in the article, Dataset S1, and *SI Appendix*.
