# Supplementary material for: Hidden proteome of synaptic vesicles in the mammalian brain
Source: Proc Natl Acad Sci U S A. 2020 Dec 21;117(52):33586–96. doi: 10.1073/pnas.2011870117 (PMC7776996; doi:10.1073/pnas.2011870117)
Supplement: Supplementary File [file pnas.2011870117.sapp.pdf]

PNAS
www.pnas.org

Supplementary Information for

Hidden Proteome of Synaptic Vesicles in the Mammalian Brain

Zacharie Taoufiq *, Momchil Ninov, Alejandro Villar-Briones, Han-Ying Wang, Toshio Sasaki, Michael C. Roy, Francois Beauchain, Yasunori Mori, Tomofumi Yoshida, Shigeo Takamori, Reinhard Jahn*, and Tomoyuki Takahashi*.

* To whom correspondence may be addressed zacharie.taoufiq@oist.jp, rjahn@gwdg.de, or ttakahas@oist.jp

This PDF file includes:

Supplementary text
Figures S1 to S8
Tables S1 to S5
SI References

Other supplementary materials for this manuscript include the following:

Dataset 'Canonical and Diversified SV-Synapse Proteome database'

Supplementary Information Text

SI Materials and Methods

Purification of synapses and synaptic vesicles from rat brain:

Synaptosomes (P2') and synaptic vesicles (SV) were purified from 4-6 weeks old rat brains. All steps were performed at 4° C. Nine brains were dissected out and homogenized in 60 mL of ice-cold sucrose buffer (0.32M sucrose, 4 mM HEPES NaOH, pH 7.4, supplemented with 1 µg/mL pepstatin and 0.2 mM PMSF protease inhibitors) in a glass-teflon homogenizer using 9 up-and-down strokes at 900 rpm. The brain homogenate (BH) was centrifuged 10 min at 2,700 rpm 4° C (Sorvall SS34 rotor). The resulting pellet (P1: cell debris, nuclei) was discarded, while the supernatant (S1) was collected and centrifuged for 15 min at 10,000 rpm 4° C (Sorvall SS34 rotor) to obtain the cytosolic fraction (S2) and the crude synaptosomal fraction (P2). A Ficoll gradient was prepared in sucrose buffer with the following layers (from bottom to top): 4 mL of 13% Ficoll, 1 mL of 9% Ficoll, and 4 mL of 6% Ficoll. The P2 fraction (3 mL in each tube) was layered over the Ficoll gradient and centrifuged for 35 min at 22,500 rpm 4° C (SW41 rotor, Beckman). The fraction at the interface between the 13% and the 9% Ficoll layers was collected, diluted in sucrose buffer and centrifuged for 12 min at 11,000 rpm 4° C (Sorvall SS34 rotor). The pellet was then resuspended in sucrose buffer to obtain the synaptosomes fraction (P2'). For SV purification, P2 suspension was additionally centrifuged for 15 min at 10,500 rpm 4° C (Sorvall SS34 rotor), and pellet was resuspended in 13 mL of sucrose buffer. This suspension, referred to as 'well-washed' crude synaptosomal fraction, was then transferred to a glass-teflon homogenizer. Osmotic lysis was immediately performed by adding 117 mL of ice-cold water and 3 up-and-down strokes at 3,000 rpm. The resulting synaptosomal lysate was buffered with 1 mL of 1 M HEPES-NaOH (pH 7.4) and centrifuged 20 min at 16,500 rpm 4° C (Sorvall SS34 rotor) to yield a lysate pellet (LP1, synaptic membranes-enriched fraction) and a lysate supernatant (LS1, cytoplasmic content of synapses). LS1 was then collected, transferred to 12 10-mL polycarbonate tubes and centrifuged for 2 hrs at 50,000 rpm 4° C (50Ti rotor, Beckman). The supernatants (LS2) were removed, and the pellets (LP2, 'crude synaptic vesicles' fraction) were resuspended in 3 mL of 40 mM sucrose. The suspension was then layered on top of a continuous 2%-22% (w/v) sucrose gradient in 5 mM HEPES pH 8.0 (generated using an automatic gradient mixer, Biocomp Instruments), and centrifuged for 4 hrs at 25,000 rpm 4° C (SW28 rotor, Beckman). The fractions corresponding to synaptic vesicles-enriched sucrose regions (= material banding at 7%-13% sucrose) were collected, pooled, and layered on top of a controlled-pore glass chromatography column (2 cm internal diameter x 150 cm) (glass beads: mean pore diameter of 300 nm, 74-125 µm (120/200 mesh) in size). The size exclusion chromatography was performed overnight in glycine buffer (0.3 M glycine, 5 mM HEPES-NaOH pH 7.2, 0,02% sodium azide) at a flow rate of 40 mL/hr. Fractions containing predominantly heterogeneous membranes with diameters exceeding 100 nm were excluded. Fractions containing uniformly shaped small vesicles with 40-45 nm diameter were then collected and centrifuged for 1 hr at 50,000 rpm 4° C (SW50.1 rotor, Beckman) to obtain the 'pure synaptic vesicles' fraction (SV). The quality of all P2' and SV purification procedures was controlled by western blots of synaptic protein markers and by electron microscopy (see '*Western blotting characterization*' and '*Electron microscopy imaging*').

Protein extraction and immunoblotting characterization:

Proteins were extracted using a lysis buffer containing 100 mM Tris-HCl (pH=8), 4% SDS, 100 mM DTT, and protease inhibitor cocktail (Sigma). Protein concentrations were analyzed by using both NanoDrop™ 2000 (Thermo Scientific) and Direct Detect® (Millipore) spectrophotometers. Proteins from each fraction were loaded equally (10 µg in each lane) onto 4-12% Bis-Tris SDS gel (NuPAGE™, Thermo Scientific), and transferred to a supported nitrocellulose membrane (Bio-Rad). The transferred membrane was blocked with 5% Skim Milk TBST for 1 hr at room temperature. All primary antibodies (see *Antibodies Table*) were used in 1% Skim Milk TBST at dilution 1:1000 and incubated with membranes overnight at 4° C. After three washes in TBST, secondary antibody-HRP conjugate was used at dilution 1:2000.

After washes, blots were developed using Clarity™ Western ECL Substrate (Bio-Rad Laboratories) and imaged on ChemiDoc™ XRS+ system with Image Lab™ Software (Bio-Rad Laboratories).

Targeted proteomics analyses:

Peptide selection criterions
We selected peptide sequences for targeted proteomics according to the following criterions: 1) unique peptide from synaptic protein detected by HD and/or UD proteomics; 2) peptide which did not include any amino acid modification such as acetylation or carbamidomethylation; 3) peptide with less than 16-amino-acid-length which allowed rapid synthesis at high purity without additional liquid chromatography purification steps.

Peptide synthesis
The peptides were synthesized through conventional 9-fluorenylmethyloxycarbonyl (Fmoc) solid-phase peptide synthesis (SPPS), onto preloaded Fmoc-$^{13}C_6{}^{15}N_2$lysine or Fmoc-$^{13}C_6$ $^{15}N_4$arginine TCP-resins (Intavis Bioanalytical Instruments), on a 1 µmole scale in 96-well plate and using a high-throughput automated peptide synthesizer ResPep SL (Intavis Bioanalytical Instruments). All Fmoc-amino acids were purchased from Watanabe Chemical Industries and prepared at 0.5 M in N-methyl pyrrolidone (NMP, Wako Pure Chemical Industries). For the automated synthesis, the following reagents (Wako Pure Chemical Industries) were dissolved in N,N-dimethylformamide (DMF) and used according to Intavis Bioanalytical Instruments SPPS protocol: 0.5M HBTU coupling reagent, 44% N-methyl-morpholine (NMM) base, 5% acetic anhydride capping reagent, and 20% piperidine deprotecting reagent. After synthesis, peptides were cleaved with (v/v/v) 92.5% TFA, 5% TIPS and 2.5% water for 2 hrs, precipitated using t-butyl-methyl-ether at -30° C, pelleted and resuspended in water before lyophilization (EYELA FDS-1000) overnight. Before use, weighted peptides were re-dissolved in water and their concentration was confirmed using a Direct Detect®infrared spectrophotometer (Millipore), the calibration curve of which was generated with peptide standards (6 x 5 LC-MS/MS Peptide Reference Mix, Promega). All synthesized peptide purity and sequence were then confirmed by LC-MS/MS (see '*Mass spectrometry*') and information on their elution time, m/z value, major charge state, and fragmentation information were collected using parallel reaction monitoring (PRM).
De novo sequencing and correlation with isotope-labeled reference
To confirm the presence of novel SV proteins in samples, peptide de novo sequencing analysis results from MS/MS data and correlation with the synthetic peptides (spectrum annotations, ion match tables, plotted mass errors) were performed using PEAKS software (v 7.0, Bioinformatics Inc).

Targeted proteomics analyses
The PRM acquisition method combined two scan events corresponding to one full scan and one PRM event targeting the doubly and triply charged precursor ions of the synthesized peptides (154 reaction masses). The full scan event employed a mass range from 350-1200 m/z, an orbitrap resolution of 70,000, a target automatic gain control (AGC) value of 5E5, and a maximum injection time of 30 ms in profile mode. The full scan event was followed by a PRM with 3 multiplexed scan events, which employed an orbitrap resolution of 17,500, a target AGC value of 1E6 and a maximum injection time of 50 ms. The precursor ion of each targeted peptide was isolated using a 1.6-m/z unit window and a positive offset of 0.4-m/z. Fragmentation was performed with stepped collision energy of 15, 22, 27 and MS/MS scans were acquired with a starting mass of 300 m/z, the ending mass being automatically defined by the charge state of the precursor ion. The generated MS/MS scan libraries were uploaded into Skyline software (version 3.6.0.10493) (1) and all the assigned fragment ions were extracted. For each SRM chromatogram, the automatic peak integrations and the native over heavy abundance ratio within peak boundaries were calculated using the software.

Absolute quantification
Absolute quantification was performed by mixing known quantities of synthetic heavy peptides (0.1 or 0.05 µg) with 50 µg of digested SV proteins. The absolute quantities of the native

peptides were calculated using the abundance signal from their corresponding heavy peptides. The absolute quantity (µg) = (0.1 or 0.05) x (native/heavy abundance ratio). Average copy number of a protein per SV was estimated using synaptotagmin 1 (Syt1), a transmembrane SV-resident protein, as a reference (15 Syt1 per SV, estimated by Takamori *et al*): Protein #/SV = absolute quantity/Molecular Weight x [(Syt1 #/SV) x (Syt1 Molecular Weight) / (Syt1 absolute quantity)].

Protein annotations in the SV proteome dataset:

For each protein identified in the SV fraction, a search in the scientific literature (PubMed, Google Scholar) and the databases (UniprotKB, NCBI) was performed manually. The most significant biological function(s) were reported, and functional keyword(s) were assigned for data filtering purpose. The structural annotations related to protein-membrane interactions were made using UniprotKB. Proteins were referred to as having transmembrane domain(s) (TM) or not, and for membrane-anchored proteins, the type of lipidation was indicated. A comparison between our data and the previously described SV proteome by Takamori *et al* was performed. As multiple names designating a given protein are frequently encountered in protein taxonomy and in the scientific literature (e.g. the tumor protein p63-regulated gene 1-like protein (Tprg1l) is also known as being Mossy fiber terminal-associated vertebrate-specific presynaptic protein (Mover) and Family with sequence similarity 79 member A protein (Fam79A)), we proceeded to rigorous cross-comparisons through amino acid sequence retrieval from the listed gi numbers and NCBI RefSeq by Takamori *et al* and the Uniprot ID-associated amino acid sequence and gene symbol from our SV protein data set. Proteins were then referred to as having been previously detected in Takamori *et al* or not. The disease annotations were made using the Uniprot and GeneCards databases for human diseases. Proteins detected in the SV fraction having 'disease(s) caused by mutation(s) affecting the gene represented in the entry' were indicated. The descriptions of syndromes were used to categorize the reported diseases into cognitive, motor and/or sensory neurological disorders. Additionally, we searched in the recently published Synaptic Gene Ontologies resource SynGO (2) for potential contaminations by postsynaptic-specific proteins in the SV fraction. Proteins were then referred to as being reported in SynGO or not and SynGO cellular components were indicated.

'Word cloud' representation:

A script was used in Python to generate a list of iBAQ values and Uniprot names of the 400 most abundant proteins in SV-1 experiment, each normalized by the iBAQ value of synaptophysin (rank 1st in SV-1). The list was uploaded into the web interface of an online word cloud generator WordArt (formerly TagUl) to generate the SV proteome word cloud image.

Amino acid sequences alignment:

The amino acid sequences of proteins were retrieved from Ensembl and aligned using the ClustalW2 program at EMBL-EBI. Alignment output and shading of the amino acids were processed using the Boxshade program (ExPASy Bioinformatics Resource Portal).

Electron microscopy imaging:

Synaptosome (P2') samples
Purified synapses were resuspended and fixed for 30 min at room temperature in 2.5% glutaraldehyde, 0.1M cacodylate buffer. After three times washes with 0.1M cacodylate buffer and centrifugations for 5 min 13,000 rpm (Eppendorf Centrifuge 5418), synapses were stained with 1% osmium 0.1M cacodylate buffer for 30 min and centrifuged 5 min 3,000 rpm (Eppendorf Centrifuge 5810 R). Synapses were then washed three times with 2 ml of pure water (Otsuka Distilled Water), and centrifuged 5 min 3,000 rpm, before successive dehydration steps in ethanol 70%, 80%, 90%, 95%, and three times in ethanol 100%. EPON resin solution was prepared on the day prior to use by mixing for 12 hrs TAAB 812, DDSA, MNA and DMP30 (TAAB Laboratories) in a ratio of 20:10:10:1, respectively. The resin solution was then mixed in a 1:1 ratio with 100% ethanol and used to resuspend and incubate synapses for 30 min at

room temperature. After centrifugation as above, synapses were resuspended in the resin solution and mixed thoroughly for 10 min. A centrifugation for 1 min 1,000 rpm and incubations in a low vacuum chamber for 1 hr and overnight on bench were performed to remove micro-air-bubbles. Samples were then centrifuged for 30 min 5,000 rpm to collect a maximum of synapses on one edge of the resin, and put into oven for 2 days at 60° C (Electron Microscope Oven TD-700, Dosaka EM co. Ltd.). Sections of samples (50 nm thickness) were performed using a Leica UC6 ultramicrotome. Synapse slices were mounted to copper grids (HF34 Maxtaform Grids 200 mesh, Nisshin EM co. Ltd.) pretreated with 100 % acetone. Slices were then stained for 30 min with 4% uranium acetate, washed four times with pure water (Otsuka Distilled Water), and stained for 5 min with a lead solution (lead nitrate (II) 1%, lead acetate trihydrate 1%, lead citrate n-hydrate 1%, from Wako Pure Chemical Industries Ltd, pH 7 adjusted with NaOH) using Nalgene® 171-0045 syringe filters and washed four times as above. Synapses images were collected on a JEM-1230R electron microscope (JEOL) operated at 100 keV and processed with Digital Micrograph software (Gatan).

Synaptic vesicle (SV) samples
Carbon-copper grids were prepared as follow: a 5-15 nm carbon film (resistance of 4 Ω/cm, purity of 99.9999%, Nisshin EM co. Ltd.) was put on copper grids (HF34 Maxtaform Grids 200 mesh, Nisshin EM co. Ltd.) using a JEOL IB-29510VET device and pretreated by glow discharge in an ion coater (DII-29020HD, JEOL) to render hydrophilic and prevent particle agglomeration during drying. Purified synaptic vesicles (3 µl, of 20 times diluted from the original sample solution, in order to observe individual vesicles) were then deposited on grids, immediately dried with paper and stained with 1% uranium acetate (phospholipids contrast agent). SV images were collected on a JEM-1230R electron microscope (JEOL) operated at 100 keV and processed with Digital Micrograph software (Gatan).

Fluorescence microscopy imaging:

SypHy and Aak1 Cloning
SypHy-P2A-TagRFP and SypHy-P2A-TagRFP-Aak1 were expressed in a neuron specific manner by using lentivirus-based vectors, in combination with Tet-Off system (3). Two vectors were used, a 'regulator' vector expressing an advanced tetracycline transactivator (tTAad) under the control of human synapsin1 promoter (STB), and a "response" vector (TGB) that expressed SypHy-P2A-TagRFP or SypHy-P2A-TagRFP-Aak1 under the control of a modified tetracycline-response element (TRE) composite promoter. To construct SypHy-P2A-TagRFP-Aak1, a full-length mouse Aak1 (accession no. NM_001040106) was amplified by PCR and subcloned into a *Stu*I site of pCR-Blunt vector (Thermo Fisher Scientific) according to manufacturer instruction and the sequence was verified. The full-length of Aak1 was excised by *Bam*HI/*Eco*RI double digestion and cloned into a *Bgl*II/*Eco*RI site of pTagRFP-C vector (Evrogen) in frame. Independently, a DNA fragment encoding sypHy lacking a stop codon (4) and a DNA fragment of a self-cleaving P2A peptide (5) were amplified by PCR and cloned into TGB vector by using In-Fusion cloning kit (Clontech) according to manufacturer instruction. Finally, a fragment encoding SypHy-P2A and that encoding TagRFP-Aak1 were PCR amplified and cloned into TGB vector by using In-Fusion cloning kit. To generate SypHy-P2A-TagRFP, essentially the same procedure was conducted except a TagRFP fragment amplified by PCR was conjugated with SypHy-P2A by using In-Fusion cloning kit.

Lentiviral-mediated expression of SypHy and Aak1
Lentivirus were produced from HEK293T cells transfected with 3.4 µg of lentiviral backbone vector (either STB or TGB with SypHy-P2A-TagRFP/TagRFP-Aak1) and helper plasmids (pCAG-kGP1 2 µg, pCAG4-RTR2 1 µg and pCAG-VSVG 1 µg) (3) using a calcium phosphate transfection method (6). Cultures were infected with STB-lentivirus at 0-1 DIV and TGB-lentivirus at 7 DIV, and subjected to experiments at 14-16 DIV.

Image analysis
Live imaging was carried out at room temperature (~24ºC) on an inverted microscope (Olympus) equipped with a 60× (1.35 NA) oil immersion objective and 75 W Xenon lamp. Images (1024 × 1024 pixels) were acquired with a cMOS camera (ORCA-Flash 4.0,

Hamamatsu Photonics) with 100 ms exposure time under the control of MetaMorph software (Molecular Devices). SypHy was imaged with 470/22 nm excitation and 514/30 nm emission filters, whereas TagRFP fluorescence was imaged with 556/20 nm excitation and 600/50 nm emission filters. Acquired images were analyzed using MetaMorph software. For quantifying TagRFP and SypHy fluorescence, line scan function in MetaMorph software was used. A Line with 5 pixels width was drawn along an axon manually, and fluorescence signals were normalized by the highest signals of either TagRFP or SypHy fluorescence in the selected region.

pHluorin-based live imaging:

Cloning

For pHluorin assay with knockdown of Aak1 expression, GFP in pLVTHM was replaced by the red fluorescent protein FusionRed (FusRed) through restriction-ligation cloning. FusRed was amplified from pCAG-FusRed template by PCR using KOD-plus-Neo DNA polymerase kit (Toyobo) adding the restriction sites MauB1 and Spe1 to the following primers: 5'-TCGACGCGCGCGGCCACCATGGTGAGCGAGCTG-3'(forward), 5'-TATGACTAGTAT TTACCTCCATCACCAG-3' (reverse). Amplified DNA fragment was run on agarose gel before purification with Monarch DNA Gel Extraction Kit (New England BioLabs), ligated to MauB1-Spe1-digested pLVTHM (T4 DNA ligase, New England BioLabs), and cloned in HST08 *E. coli* strain (Stellar™ competent cells, Clontech). Confirmed by sequencing plasmids were purified from *E. coli* using Plasmid Maxi kit (Qiagen). pLVTHM-FusRed-shRNA Lentiviruses were then prepared as described in '*Lentivirus preparation and titration'*.

Phluorin assay

Dissociated hippocampal neurons were transfected at DIV0 with 0.8 µg of pCAG-SypHy2x plasmid by electroporation (one pulse at 1360V, 24 ms, for a 10 µL suspension of 100,000 cells) using Neon Transfection System (Invitrogen). After growing, cells were infected at DIV11-12 with pLVTHM-FusRed lentiviruses expressing the shRNA (see '*shRNA cloning and knockdown assay'*). Before imaging, cells cultured on a glass coverslip (DIV15-19) were placed on the built-in imaging chamber of the confocal microscope and continuously perfused with standard extracellular solution at 25°C containing (in mM): 140 NaCl, 2.4 KCl, 10 HEPES, 10 glucose, 2 $CaCl_2$, 1 $MgCl_2$, 0.01 CNQX (pH 7.4). SypHy imaging was performed on a Zeiss LSM780 confocal microscope with a C-Apochromat 40x/1.2 W Korr M27 objective. Neurons were identified using SypHy resting fluorescence (neurons expressing synaptophluorin) and FusRed fluorescence (neurons expressing shRNA) with GFP band pass filter 488 nm excitation and 493-586 nm emission, and mCherry band pass filter 561 nm excitation and emission 578-697 nm, respectively. A concentric bipolar electrode (FHC), placed 80-100 µm away from the neuron, delivered a train of pulses (1ms, 8V) at 10Hz for 10 s. Image acquisition was carried out on a portion of axon of the stimulated neuron, in time-lapse mode, at 1-2 frames per second, through Zen software v2.1 (Zeiss). Baseline fluorescence was recorded for 1 minute before each stimulation.

Image analysis

Image analysis was performed using ImageJ (National Institutes of Health), and OriginPro2017 (OriginLab Corporation). In ImageJ, square regions of interest of 1.6µm side were positioned manually at the centre of fluorescence puncta, and the corresponding fluorescence data was extracted to Origin. Fluorescence time course of raw traces were corrected for photo-bleaching with the fitted baseline fluorescence intensity F_0 as $(F-F_0)/F_0$ at each time point by OriginPro2017. Half-decay time was measured as the time required by the signal to reach half of the peak intensity after stimulation. The average half decay time data from 51 boutons of 16 neurons over 5 independent experiments for Aak1 knockdown was compared with average half decay tine data from 20 boutons of 10 neurons over 4 independent experiments.

Electrophysiological assays:

Brain slice preparation and solutions

Wistar rats (postnatal day 13-15) of either sex were killed by decapitation under isoflurane anesthesia. Transverse brainstem slices (175-200 μm in thickness) containing the medial nucleus of the trapezoid body (MNTB) were cut in ice-cold solution containing (in mM): 200 sucrose, 2.5 KCl, 26 $NaHCO_3$, 1.25 NaH_2PO_4, 6 $MgCl_2$, 10 glucose, 3 myo-inositol, 2 sodium pyruvate, and 0.5 sodium ascorbate (pH 7.4 when bubbled with 95% O_2 and 5% CO_2, 310–320 mOsm) by using vibroslicer (VT1200S, Leica). Before recording, slices were incubated for 1 h at 37 ºC in standard aCSF solution containing (in mM): 125 NaCl, 2 KCl, 26 $NaHCO_3$, 1.25 NaH_2PO_4, 2 $CaCl_2$, 1 $MgCl_2$, 10 glucose, 3 myo-inositol, 2 sodium pyruvate, and 0.5 sodium ascorbate (pH 7.4 when bubbled with 95% O_2 and 5% CO_2, 310–320 mOsm), and maintained thereafter at room temperature (24–26 ºC). MNTB principal neurons and calyx of Held presynaptic terminals were visually identified using a x40 water immersion objective attached to an upright microscope (BX51WI, Olympus).

Membrane capacitance measurement

Membrane capacitance measurement from the calyx of Held presynaptic terminals, in whole-cell configurations, were made at room temperature (RT, 26–27 °C). Data were acquired at a sampling rate of 50 KHz, using an EPC-10 patch-clamp amplifier controlled by PatchMaster software (HEKA) after on-line filtering at 5 kHz. Calyx of Held terminals were voltage-clamped at a holding potential of -80 mV and a sinusoidal voltage command with a peak-to-peak voltage of 60 mV was applied at 1 kHz. Aak1 inhibitor LP935509 (Axon MedChem) was dissolved in DMSO (0.1 %), which was also included in pipette solution, and infused from whole-cell pipettes into calyceal terminals by diffusion. Care was taken to keep the access resistance below 14 MΩ to allow diffusion of the drug into the terminal within 5 min after whole-cell rupture. To isolate presynaptic voltage-gated calcium charge transfer (Q_{Ca}), the aCSF contained 10 mM tetraethylammonium chloride (TEA, Tokyo Chemical Industry Ltd.), 0.5 mM 4-aminopyridine (4-AP, Nacalai Tesque), 1 mM tetrodotoxin (TTX, Nacalai Tesque), 10 mM bicuculline methiodide (Santa Cruz Biotechnology) and 0.5 mM strychnine hydrochloride (Tokyo Chemical Industry Ltd.). Intracellular solution for presynaptic terminals contained (in mM): 125 Cs-methanesulfonate, 30 CsCl, 10 HEPES, 0.5 EGTA, 12 Na_2-phosphocreatine, 3 MgATP, 1 $MgCl_2$, 0.3 Na_2GTP (315-320 mOsm, pH 7.3 adjusted with CsOH). Tips of recording pipettes were coated with dental wax (GC Corporation) to reduce stray capacitance (4-6 pF). Single-pulse step depolarization to +10 mV for 20 ms was used to induce presynaptic Q_{Ca}. Membrane capacitance (C_m) changes within 450 ms after square-pulse stimulation were excluded from analysis to avoid contamination with conductance- dependent capacitance artifacts. Data were obtained within 20 min after whole-cell rupture. The amplitude of exocytic C_m change (ΔC_m) was measured as the difference of C_m values between the baseline and those at 450–500 ms after depolarization. Sample C_m records are shown as average values of each 50-data point (for 50 ms) plotted every 50 ms (for shorter time scale) or every 500 ms (for longer time scale). The half decay time of endocytosis was measured from the midpoint of ΔC_m decay.

EPSC recording

For recording of evoked EPSCs, simultaneous pre- and postsynaptic whole-cell recordings were made from a calyceal nerve terminal and postsynaptic cell. Throughout the experiments, presynaptic recordings were made in current-clamp mode, whereas postsynaptic recordings were made in voltage-clamp mode at a holding potential of -70 mV. Pipette solution for recording of presynaptic action potentials (APs) contained (mM): 110 K-gluconate, 10 L-glutamate, 30 KCl, 10 HEPES, 0.5 EGTA, 12 Na_2-phosphocreatine, 3 MgATP, 1 $MgCl_2$, 0.3 Na_2GTP (315 mOsm, pH 7.3 adjusted with KOH), and that for postsynaptic recording contained (mM): 110 CsF, 30 CsCl, 10 HEPES, 5 EGTA, 1 $MgCl_2$, 5 QX314-Cl (300 mOsm, pH 7.3 adjusted with CsOH). EPSCs were evoked by current injection (0.5-1 nA, 1 ms) into the presynaptic terminal via a recording glass electrode, in the presence of bicuculline methiodide (10 μM) and strychnine hydrochloride (0.5 μM).

Action potential recordings

For recording postsynaptic action potentials (APs), simultaneous pre- and postsynaptic whole-cell recordings were made from calyceal terminals and postsynaptic MNTB principal neurons, both in current-clamp mode, in the presence of bicuculline methiodide (10 μM) and strychnine

hydrochloride (0.5 µM). Pipette solution for postsynaptic AP recording contained (mM): 120 K-gluconate, 30 KCl, 5 EGTA, 12 Na_2-phosphocreatine, 3 MgATP, 1 L-arginine, 1 $MgCl_2$, 0.3 Na_2GTP (315 mOsm, pH 7.3 adjusted with KOH). Presynaptic APs were elicited by a square pulse current injection into calyces in current-clamp mode, via recording glass electrodes filled with K-gluconate-based internal solution (as above).

Data statistical analysis
Data were analyzed using IGOR Pro 6 (WaveMetrics), Excel 2011 (Microsoft) and SigmaPlot 12 (Systat Software Inc.). All values are given as mean ±S.E.M., and $p < 0.05$ was taken as a significant difference in Student's *t*-test, one-way ANOVA with the Bonferroni *post-hoc* test.

Hippocampal cell culture electrophysiology
Whole-cell patch-clamp recordings were made from dissociated hippocampal cultures at DIV15 (earliest time for a complete Aak1 knockdown in neuronal cells infected by lentiviruses at DIV11-12, and culture developmental stage from which endogenous expression of major SV protein synaptophysin is detected (Figure S6A)). The pipette solution contained (in mM): 110 CsF, 30 CsCl, 10 HEPES, 5 EGTA, 1 $MgCl_2$, 5 QX314-Cl (300 mOsm, pH 7.3 adjusted with CsOH). Cells were continuously perfused with standard aCSF solution containing (in mM): 125 NaCl, 2 KCl, 26 $NaHCO_3$, 1.25 NaH_2PO_4, 2 $CaCl_2$, 1 $MgCl_2$, 10 glucose, 3 myo-inositol, 2 sodium pyruvate, and 0.5 sodium ascorbate (pH 7.4 when bubbled with 95% O_2 and 5% CO_2, 310–320 mOsm) with 0.05 D-AP5, 0.01 bicuculline methiodide. The stimulating bipolar electrode was positioned close to the afferent neuron ~80-100 µm distant from the target neuron visualized with GFP under fluorescence microscope. Neurons were voltage clamped at −70 mV with an EPC-10 amplifier (HEKA Electronics, Germany). Only cells with series resistances of <15 MΩ, with 70–80% of this resistance compensated, were analyzed. Currents were acquired using PATCHMASTER software (HEKA Electronics), filtered at 5 kHz, and digitized at 10 kHz. Data were analyzed using AxographX (Axograph Inc., USA), and IgorPro (WaveMetrics Inc., USA). All experiments were carried out at room temperature. All values are given as mean ± S.E.M., and $p < 0.05$ was taken as a significant difference in Student's *t*-test, paired *t*-test, one-way ANOVA with the Bonferroni *post-hoc*.

Dissociated hippocampal cell culture:

Primary hippocampal cell cultures were performed following the description from (7). Neonatal pups (P1) of mice (ICR CD-1, Charles River Laboratories) were sacrificed by decapitation and hippocampi were dissected out at 4° C in filter-sterilized HBSS buffer containing 0.1% glucose (Thermo Scientific), 1 mM sodium pyruvate (Thermo Scientific), and 10 mM HEPES (Sigma). Hippocampal tissue was then digested using the papain-based Neuron Dissociation Solutions S kit (Wako Pure Chemical Industries Ltd). Harvested cells were plated on poly-L-lysine coated µ-Dish 35 mm low (Ibidi) with Basal Medium Eagle (Thermo Scientific) containing 0.45% glucose, 1 mM sodium pyruvate, 2 mM L-glutamine (Thermo Scientific), and supplemented with 10% (v/v) fetal bovine serum (Thermo Scientific) and placed at 37° C under 5% CO_2. After 3 hrs, the plating medium was replaced by maintenance medium: Neurobasal™ Medium (Thermo Scientific) containing 2 mM L-glutamine, and 2% (v/v) B27™ Supplement (Thermo Scientific). At 4 days in vitro, half of the culture medium was replaced by freshly prepared maintenance medium and replaced similarly every 3 or 4 days.

HEK293T cells:

Human embryonic kidney (HEK) 293T cells (Lenti-X™ 293T Cell Line, Clontech) were seeded at 25% confluence on 100 mm BioCoat™ Collagen I pre-coated culture dishes (Corning).and cultured in DMEM high glucose (Thermo Scientific) containing 1 mM sodium pyruvate (Thermo Scientific) and 10% (v/v) fetal bovine serum (Biological Industries) at 37° C under 5% CO_2. Cells were passaged at 90% confluence by trypsinization and reseeded.

Lentivirus preparation and titration:

When reaching 90% confluence, HEK293T cell dishes were each transfected with 7.3 µg of pLVTHM (transfer plasmid containing the shRNA of interest and a GFP reporter gene (8), 5 µg of psPAX2, and 2.3 µg of pMD2.G (lentivirus packaging plasmids, gifts from Didier Trono, Addgene plasmids # 12247, # 12260, and # 12259 respectively), using 75 µg of Polyethylenimine Max 40K (Polysciences Inc.) in 1 mL Opti-MEM™ (Thermo Scientific) added to the culture dish. Cells were incubated for 7 hrs at 37° C under 5% CO_2, then media were replaced with 8 mL of fresh culture media. Cell culture supernatants (containing lentiviruses) were collected after 48 hrs, and filtered through 0.45 µm syringe filter, before ultracentrifugation for 2 hrs 87,000 g at 4° C (JS-24.15 rotor, Beckman Coulter). Lentiviral pellets were then resuspended with PBS, placed on ice for 2 hrs, aliquoted (3 x 5 µL of lentiviral suspensions from each HEK293T cell culture dish), and stored at -80°C.
To titrate the pLVTHM-based lentiviruses, primary hippocampal cultures at days *in vitro* (DIV)6 were used and infected with serial dilutions (1:1,000 to 1:500,000) in fresh maintenance medium of a frozen lentiviral aliquot. After 24 hrs, the medium containing lentiviruses was replaced by fresh maintenance medium. Infected cultures dishes were then observed at DIV11-12 with a confocal microscope (LSM 780, Zeiss). Images were acquired and analyzed with brightness and contrast set at the limit of appearance of autofluorescence on the control uninfected dish. The total number of neurons and GFP positive neurons were counted to calculate the biological titer (BT) in transducing units (TU) per mL according to the formula described in (9): BT = (F x N x D)/V; where F is the percentage of GFP positive cells, N is the number of cells counted in the dish, D is the dilution factor, and V is the volume in mL of dilution. All lentiviruses used in the experiments presented a biological titer above $3x10^9$ TU/mL at dilution 1:1,000 with a GFP positive infection rate > 95%.

shRNA cloning and knockdown assay:

RNA interference knockdown was performed by plasmid-based short hairpin RNA (shRNA) using the sequence 5'-CAGTCAACCTCTTCAGTCA-3', which targets efficiently mouse Aak1 at nucleotide position 1808-1826 (Exon 11, previously used and described in (10). The sequence 5'-ACCCTATTCCTGTACTAATTA-3'targeting a region in exon 20 of Aak1 and which did not show any interference effect on Aak1 expression in our western blot experiments was used as a negative control (shRNA-control). The specific forward and reverse shRNA oligonucleotides, flanked by restriction sites Mlu1 and Cla1, were designed to contain the sense strand of 19 or 21 nucleotide target sequence, followed by a short spacer (TTCAAGAGA), and the reverse complement of the sense strand. Five thymidines were added at the end of the oligonucleotide as RNA transcriptional stop signal (see table for full custom sequences). Customized oligos (Fasmac) were annealed at 2 µM in annealing buffer (OriGene) at 95° C for 5 min, followed by incubation at 70° C for 10 min and slow cooling to room temperature. Annealed oligos were then inserted into pLVTHM lentiviral vector digested by Mlu1 and Cla1, downstream to the H1 promoter. All constructs were confirmed by sequencing service from Fasmac. Hippocampal neuron cultures at DIV11-12 were infected with GFP and shRNA-expressing lentiviruses (see '*Lentivirus preparation and titration*'). At DIV15, neuronal cells were scraped, proteins extracted, and the expressions of GFP (lentiviral infection reporter) and aak1 (RNAi target) were monitored by western blot (see '*Western blot characterization*').

Proteomic data repository

The proteomic raw data files of this study are available through the Japan Proteome Standard Repository Database (11). The accession numbers are PXD021549 for ProteomeXchange and JPST000968 for jPOST.

Resources and Reagents:

ANTIBODIES	SOURCE	REFERENCE NUMBER
Primary antibodies		
Guinea pig anti-gephyrin	Synaptic Systems	147004
Mouse anti-α-tubulin	Sigma	T6199
Mouse anti-GRP-75	Abcam	ab2799
Mouse anti-munc 13-1	Synaptic Systems	126111
Mouse anti-neuroligins 2/3	Synaptic Systems	129211
Mouse anti-NMDA-R1	Reinhard Jahn's laboratory	clone 54.3
Mouse anti-SNAP-25	Reinhard Jahn's laboratory	clone 71.1
Mouse anti-SV2A	Synaptic Systems	119011
Mouse anti-SV2B	Synaptic Systems	119111
Mouse anti-synaptobrevin 2	Reinhard Jahn's laboratory	clone 69.1
Mouse anti-synaptogyrin 1	Reinhard Jahn's laboratory	clone 80.1
Mouse anti-synaptophysin	Synaptic Systems	101011
Mouse anti-synaptotagmin 1	Reinhard Jahn's laboratory	clone 41.1
Mouse anti-syntaxin-1	Synaptic Systems	110011
Rabbit anti-Aak1	Abcam	ab59740
Rabbit anti-calcium channel P/Q-type (α-1A)	Synaptic Systems	152103
Rabbit anti-caskin 1	Synaptic Systems	185002
Rabbit anti-CB1	Synaptic Systems	258003
Rabbit anti-cox-4	Synaptic Systems	298002
Rabbit anti-CSPα	Synaptic Systems	154003
Rabbit anti-Fbxo41	Synaptic Systems	332003
Rabbit anti-fis1	Genetex	GTX111010
Rabbit anti-GAPDH	Abcam	ab9485
Rabbit anti-γ-enolase	Synaptic Systems	230002
Rabbit anti-GFP	Abcam	ab290
Rabbit anti-homer 1	Synaptic Systems	160002
Rabbit anti-liprin-α-3	Synaptic Systems	169002
Rabbit anti-MARK2	Synaptic Systems	331003
Rabbit anti-mint-1	Synaptic Systems	144103
Rabbit anti-mover	Synaptic Systems	248003
Rabbit anti-N-cadherin	Synaptic Systems	363003
Rabbit anti-PSD-95	Synaptic Systems	124002
Rabbit anti-pygb	Synaptic Systems	255002
Rabbit anti-renin receptor	Thermo Scientific	PA5-28440
Rabbit anti-rims 1/2	Synaptic Systems	140203
Rabbit anti-SV31	Synaptic Systems	228002
Rabbit anti-synaptogyrin 3	Synaptic Systems	103302
Rabbit anti-TNiK	Synaptic Systems	290003
Rabbit anti-veli	Synaptic Systems	184002
Rabbit anti-VGLUT1	Synaptic Systems	135303
Rabbit anti-vMAT2	Synaptic Systems	138302
Secondary antibodies		
Goat anti-guinea pig IgG-HRP conjugate	Novex	A18769
Goat anti-mouse IgG-HRP conjugate	Bio-Rad	1706515
Goat anti-rabbit IgG-HRP conjugate	Bio-Rad	1721011

REAGENT or RESSOURCE	SOURCE	REFERENCE NUMBER
Chemicals		
Clarity™ Western ECL Substrate	Bio-Rad	170-5060
NuPAGE™ 4-12% Bis-Tris Gels, 1.0 mm, 10-well	Thermo Scientific	NP0321
Protease Inhibitor Cocktail	Sigma	MSSAFE
Pall Nanosep® 10K Omega	Sigma	Z722073
Lys-C	Promega	V1671
Trypsin/Lys-C	Promega	V5071
Fmoc-13C6 15N2 lysine TCP-resin	Intavis Bioanalytical Instruments	PC-01-0318
Fmoc-13C6 15N4 arginine TCP-resin	Intavis Bioanalytical Instruments	PC-01-0326
6 x 5 LC-MS/MS Peptide Reference Mix	Promega	V7491
LP935509	Axon MedChem	2638
Experimental models: animal		
rats	Charles River Laboratories	Sprague Dawley
mice	Charles River Laboratories	strain Hsd:ICR(CD-1®)
Experimental models: cell line		
HEK293T cells	Clontech	Lenti-X™ 293T Cell Line (#632180)
Plasmids		
pLVTHM	Laboratory of Virology and Genetics (Didier Trono)	Addgene # 12247
psPAX2	Laboratory of Virology and Genetics (Didier Trono)	Addgene # 12260
psMD2.G	Laboratory of Virology and Genetics (Didier Trono)	Addgene # 12259
pCAG-kGP1	Hioki et al., 2009	*N/A*
pCAG4-RTR2	Hioki et al., 2009	*N/A*
pCAG-VSVG	Hioki et al., 2009	*N/A*
STB	Hioki et al., 2009	*N/A*
shRNA oligonucleotides primers		
shAak1 (forward) 5'-CGCGTCCCCCAGTCAACCTCTTCAGTCAttcaagagaTGACTGAAGAGGTTGACTGTTTTTGGAAAT-3'		
shAak1 (reverse) 5'-CGATTTCCAAAAACAGTCAACCTCTTCAGTCAtctcttgaaTGACTGAAGAGGTTGACTGGGGGA-3'		
shRNA-control (forward) 5'-CGCGTCCCCACCCTATTCCTGTACTAATTAttcaagagaTAATTAGTACAGGAATAGGGTTTTTTGGAAAT-3'		
shRNA-control (reverse) 5'-CGATTTCCAAAAAACCCTATTCCTGTACTAATTAtctcttgaaTAATTAGTACAGGAATAGGGTGGGGA-3'		
Software and Algorithms		
Proteomics		
Proteome Discoverer™ version 2.2	Thermo Scientific	*N/A*
Mascot version 2.6	Matrix Science	*N/A*
Sequest HT	Thermo Scientific (licence), University of Washington	*N/A*
Skyline 3.6.0.10493	MacCoss Lab Software, University of Washington	*https://skyline.ms/project/home/begin.view?/*
PEAKS version 7.0	Bioinformatics Solutions Inc.	*N/A*
Statistical analyses		
R version 3.2.5	The R Project for Statistical Computing	*www.r-project.org*
Imaging		
Digital Micrograph™	Gatan	*N/A*
Zen software v2.1	Zeiss	*N/A*
ImageJ	*N/A*	*N/A*
MetaMorph	Molecular Devices	*N/A*
Web interface / server		
Tagul	WordArt	*https://tagul.com or https://wordart.com*
UniprotKB *Rattus norvegicus*	Uniprot.Org	Proteome ID: UP000002494
Boxshade	ExPASy Bioinformatics Resource Portal	*://embnet.vital-it.ch/software/BOX_form.html*
Synaptic Gene Ontologies	SynGO consortium	*https://www.syngoportal.org/index.html*

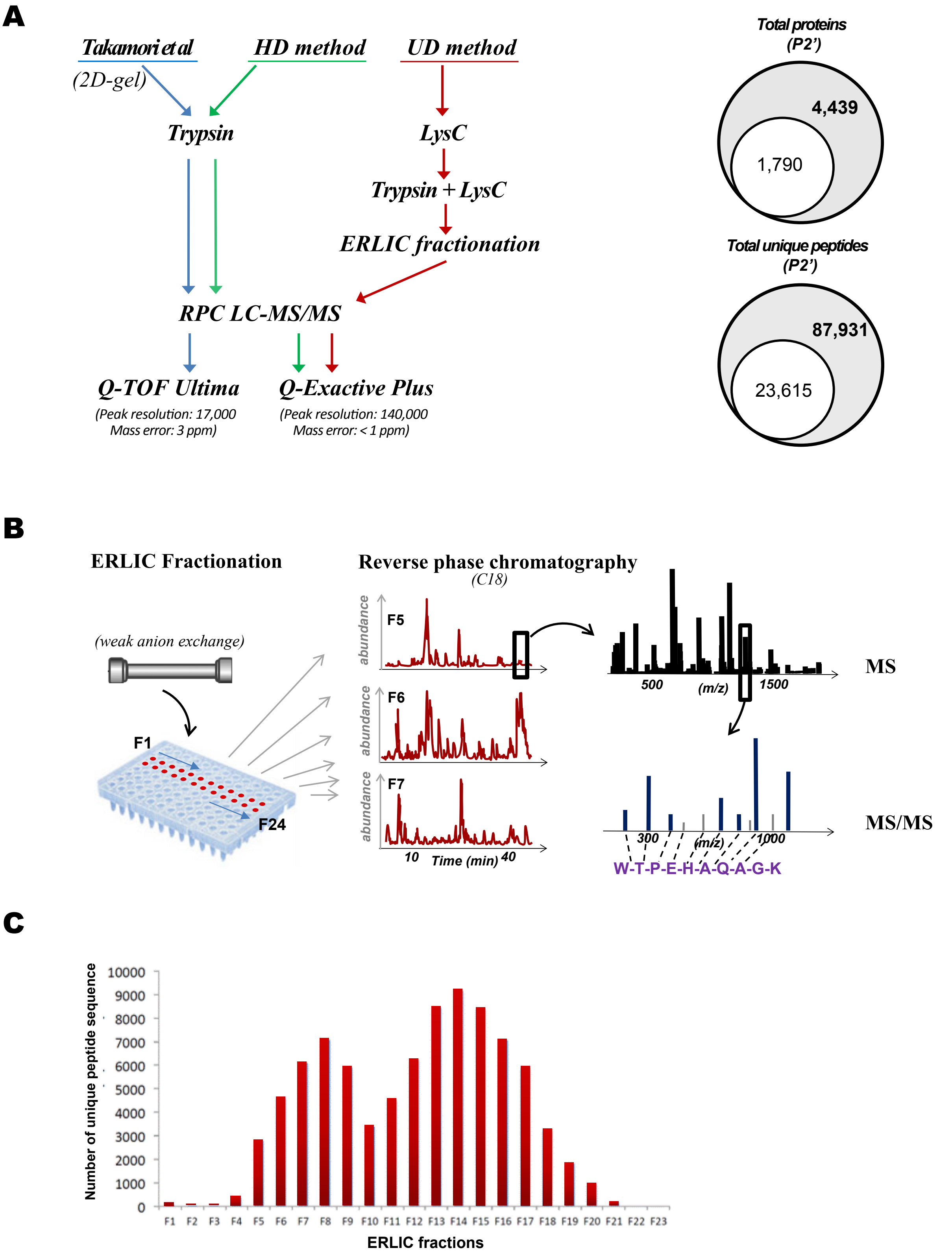

Figure S1 Methodological advancement of SV proteomics

(A) Schematic description of different protocols and MS instruments employed by Takamori *et al.* (2006), the HD method, and the UD method. Takamori *et al* and the HD method utilize

conventional one-step trypsin digestion and on-line reverse-phase chromatography (RPC), whereas the UD method utilizes sequential protein digestion steps with LysC and trypsin-LysC in combination, followed by off-line electrostatic repulsion-hydrophilic interaction chromatography (ERLIC) and on-line RPC of each of the ERLIC fractions. This protocol enabled identification of 4,439 proteins comprising 87,931 unique peptides (*right panels*) in the purified synaptosomal fraction (P2’). This outnumbers those identified by the HD methods by 2.5 and 3.7 times, respectively.

(B) ERLIC fractions resolved by C_{18}-reverse phase chromatography (RPC). Orthogonal peptide separation with a mechanism based on multiple biophysical properties of amino acids maximizes separation of peptides with similar masses, but different compositions and sequences. ERLIC separates peptides according to their charges, polarities, pIs, orientations, post-translational modifications (e.g. phosphorylation), and RPC, according to hydrophobicity. A small unmasked peak in a square contains many peptides that were analyzed by LC-MS/MS and sequenced for an in-depth proteome identification.

(C) Number and distribution of unique peptides identified across the 24 ERLIC fractions (F) in the P2’ sample. The highest numbers of unique peptides were detected in F7-9 and F12-17.

A

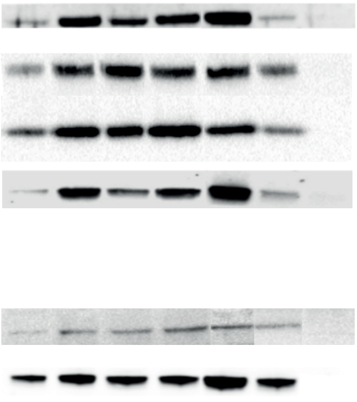

Postsynaptic proteins

	HD P2’	HD SV	UD P2’	UD SV	(Uniprot ID)
PSD-95	5.364 E8	0	4.825 E8	0	(P31016)
gephyrin	3.174 E6	0	1.101 E8	0	(Q03555)
homer 1	7.197 E7	0	2.494 E8	0	(Q9Z214)
NMDA-R1	7.251 E7	0	1.087 E8	0	(P35439)

Synaptic cleft proteins

	HD P2’	HD SV	UD P2’	UD SV	(Uniprot ID)
neuroligins 2/3	1.403 E7	0	6.014 E7	0	(Q62888)
	7.095 E7	0	8.277 E7	0	(Q62889)
N-cadherin	5.874 E7	0	1.014 E8	0	(Q9Z1Y3)

B

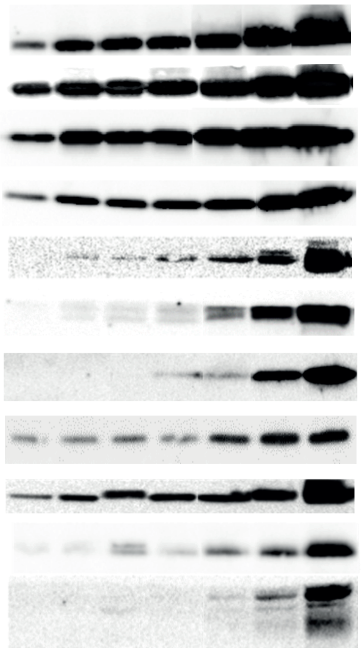

SV proteins

	HD P2’	HD SV	UD P2’	UD SV	(Uniprot ID)
synaptotagmin 1	9.813 E8	2.526 E9	1.261 E9	3.703 E9	(P21707)
VGLUT1	1.493 E8	9.153 E8	1.159 E9	4.232 E9	(Q62634)
synaptophysin	2.059 E9	1.111 E10	2.698 E9	1.175 E10	(P07825)
synaptobrevin 2	3.756 E8	6.786 E9	2.375 E9	4.360 E9	(P63045)
SV2A	1.596 E8	1.640 E9	8.533 E8	3.969 E9	(Q02563)
SV2B	2.048 E8	6.099 E8	4.514 E8	2.058 E9	(Q63564)
synaptogyrin 1	1.848 E8	9.675 E8	8.247 E8	2.677 E9	(Q62876)
synaptogyrin 3	0	4.641 E8	3.596 E8	2.877 E9	(D4ABK1)
CSPa	0	3.942 E8	2.122 E8	8.883 E8	(P60905)
SV31	0	2.556 E8	1.046 E8	3.165 E8	(A9CMA6)
VMAT2	0	3.642 E7	4.890 E6	3.370 E7	(Q01827)

C

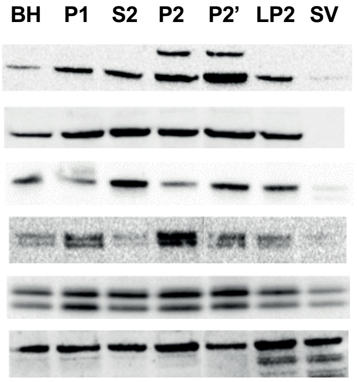

AZ proteins

	HD P2'	HD SV	UD P2'	UD SV	(Uniprot ID)
munc 13-1	5.883 E7	0	6.515 E7	7.857 E5	(Q62768)
liprin-α-3	5.724 E7	0	9.652 E7	0	(F1LSE6)
mint-1	-	-	1.203 E7	0	(O35430)
rim 1	2.535 E7	0	4.897 E7	1.889 E6	(Q9JIR4)
rim 2	-	-	4.032 E7	2.664 E6	(Q9JIS1)
veli-1	8.616 E6	0	2.315 E8	3.689 E7	(Q9Z250)
veli-3	0	5.352 E6	1.859 E8	1.938 E7	(Q792I0)
caskin 1	2.738 E7	8.979 E6	1.068 E8	1.982 E7	(D3ZE17)

D

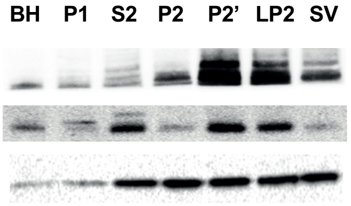

Presynaptic membrane proteins

	HD P2'	HD SV	UD P2'	UD SV	
Ca^{2+} channel P/Q type (α-1A)	-	-	5.349 E7	5.256 E6	(P54282)
CB1	-	-	6.086 E7	9.530 E6	(P20272)
SNAP-25	3.570 E8	2.160 E8	1.399 E9	5.672 E8	(P60881)

E

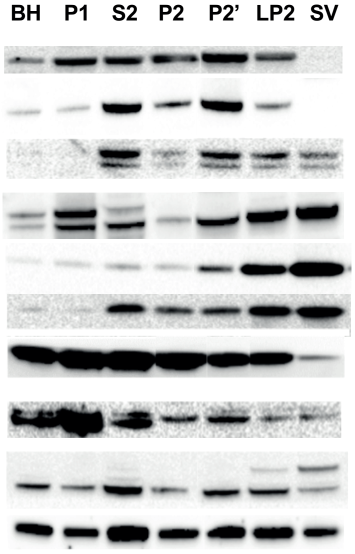

Cytoplasmic proteins

	HD P2'	HD SV	UD P2'	UD SV	
fbxo41	1.009 E8	0	4.476 E7	0	(D3ZT20)
TNiK	-	-	4.560 E7	0	(D3ZZQ0)
MARK2	0	8.487 E6	5.579 E7	2.931 E7	(O08679)
aak1	2.466 E7	2.672 E8	1.654 E8	6.875 E8	(F1LRI7)
mover	3.909 E7	3.408 E8	5.857 E7	2.299 E8	(A8WCF8)
AP-2 complex subunit mu	1.656 E8	6.537 E8	7.733 E8	2.419 E9	(P84092)
γ-enolase	1.033 E9	1.059 E7	2.578 E9	1.784 E7	(P07323)
α-tubulin	7.626 E9	1.068 E8	1.037 E10	3.601 E8	(P68370)
pygb	2.417 E8	2.275 E7	4.889 E8	3.804 E7	(G3V6Y6)
GAPDH	3.345 E9	1.715 E9	7.456 E9	2.357 E9	(P04797)

F

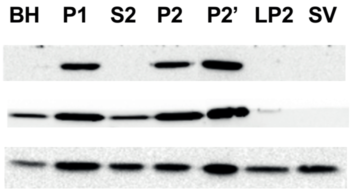

Mitochondrial proteins

	HD P2'	HD SV	UD P2'	UD SV	
GRP-75	5.166 E8	0	9.828 E8	0	(F1M953)
cox-4	4.809 E8	0	4.140 E9	0	(P10888)
fis1	1.622 E8	2.947 E7	1.104 E8	2.864 E7	(P84817)

Figure S2. UD proteomics renders synaptic proteomes quantifiable

Western blot screening of 41 synaptic proteins, compared with quantitative data obtained from HD and UD proteomic methods. Left panels: Western blot profiles of proteins in brain subcellular fractionation. Right panels: MS quantification of corresponding proteins obtained using HD or UD methods from the P2' and SV fractions. Values indicate intensity-based absolute quantification (iBAQ) of proteins in exponential notation (average of 3 independent experiments). Mismatches between western blot profiles and iBAQ (indicated by red dashed boxes) were observed in 30% (13/41) of cases with the HD method, but none with the UD method. (A) Postsynaptic and cleft, (B) SV, (C) AZ, (D) presynaptic membrane, (E) cytoplasmic, and (F) mitochondrial proteins.

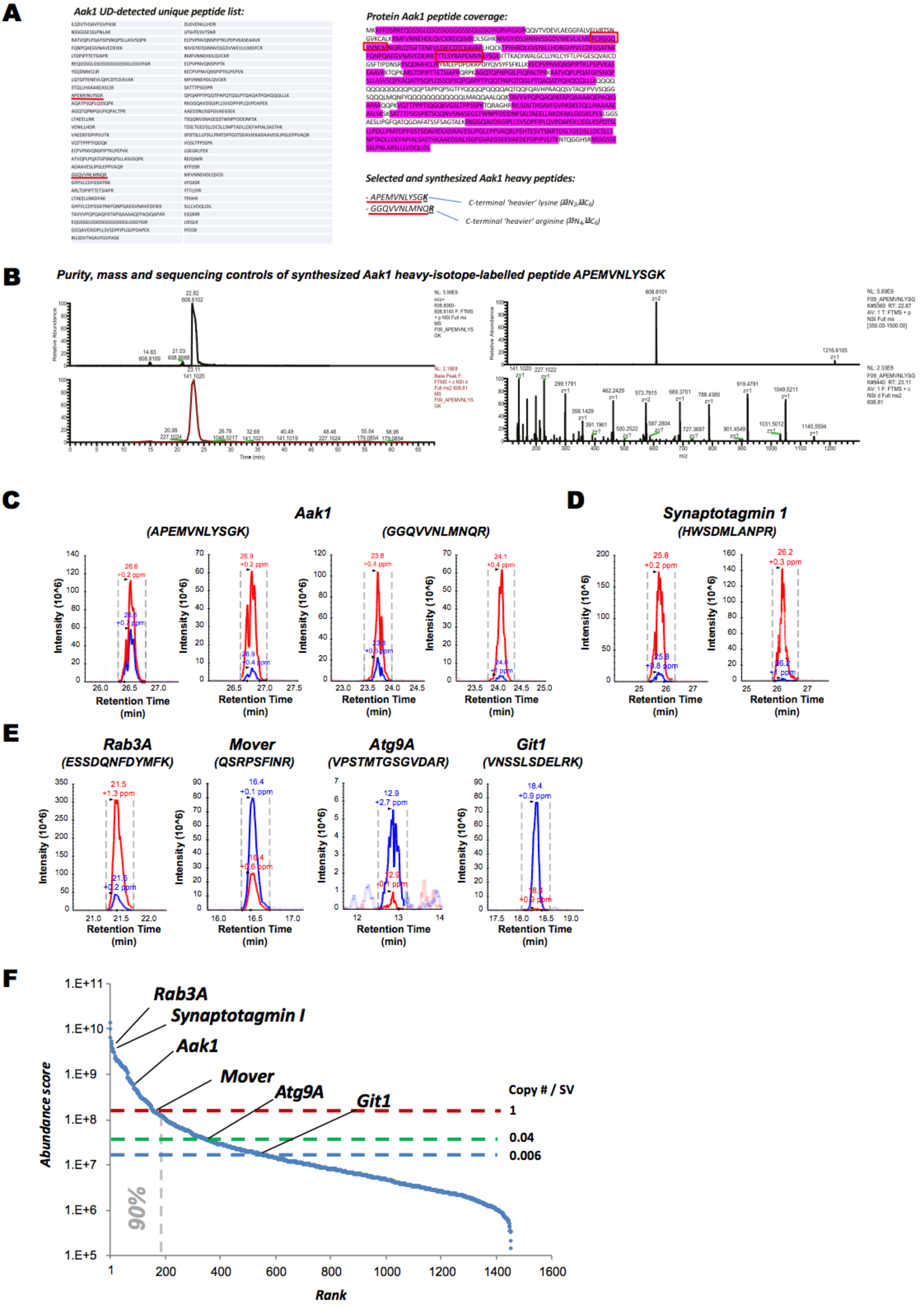
A
Aak1 UD-detected unique peptide list:
Protein Aak1 peptide coverage:
Selected and synthesized Aak1 heavy peptides:
- APEMVNLYSGK
- GGQVVNLMNQR
C-terminal 'heavier' lysine
C-terminal 'heavier' arginine
B
Purity, mass and sequencing controls of synthesized Aak1 heavy-isotope-labelled peptide APEMVNLYSGK
Relative Abundance
Time (min)
m/z
C
Aak1
(APEMVNLYSGK)
(GGQVVNLMNQR)
D
Synaptotagmin 1
(HWSDMLANPR)
Intensity (10^6)
Retention Time (min)
E
Rab3A
(ESSDQNFDYMFK)
Mover
(QSRPSFINR)
Atg9A
(VPSTMTGSGVDAR)
Git1
(VNSSLSDELRK)
F
Abundance score
Rank
Rab3A
Synaptotagmin I
Aak1
Mover
Atg9A
Git1
Copy # / SV
1
0.04
0.006
90%

Figure S3 Estimation of protein copy number per SV of the total SV proteome

(A) *(left panel)* List of Aak1 unique peptides detected by the UD method. *(right)* Detected peptides highlighted within the Aak1 amino acid sequence. Two selected peptides (underlined) were synthesized by conventional Fmoc solid-phase peptide synthesis (SPPS) using heavy lysine or arginine at the C-terminus.

(B) Purity and sequence confirmation of synthetic APEMVNLYSG*k* peptide in LC-MS/MS analyses in a full MS chromatogram *(a)*, base peak fragment ion MS2 chromatogram *(b)*, precursor *(c)* and product *(d)* ion spectra, which give elution times, m/z ratios, charge states, and fragmentation information further used to track the native peptide after mixing with digested SV proteins.

(C) Chromatograms of precursor ion MS1 of Aak1-specific heavy peptides (blue traces) superimposed upon respective native peptides (red traces). Isotope-labeled heavy peptides at two doses (0.1 µg in left panels; 0.02 µg in right panels) were mixed with each 50 µg of digested SV protein sample before LC-MS/MS analysis. Automatic peak integrations and native/heavy signal ratios within peak boundaries (vertical lines) were calculated using Skyline software (version 3.6.0.10493, Supplemental Table S1). Retention time (min) and mass error (ppm) are indicated on top of the peaks.

(D) Chromatograms of the heavy peptide and the native peptide of synaptotagmin 1 (two panels as above). These data are used as a standard for calculating copy number per SV (see experimental procedures).

(E) Chromatograms of heavy and native peptides in Rab3A, Mover, Atg9A and Git1 proteins (heavy peptides, 0.1 µg).

(F) Position of selected proteins shown on the SV proteome ranked (iBAQ) abundance curve. Horizontal dashed lines delineate protein copy numbers per SV estimations in the SV proteome (Supplemental Table S1).

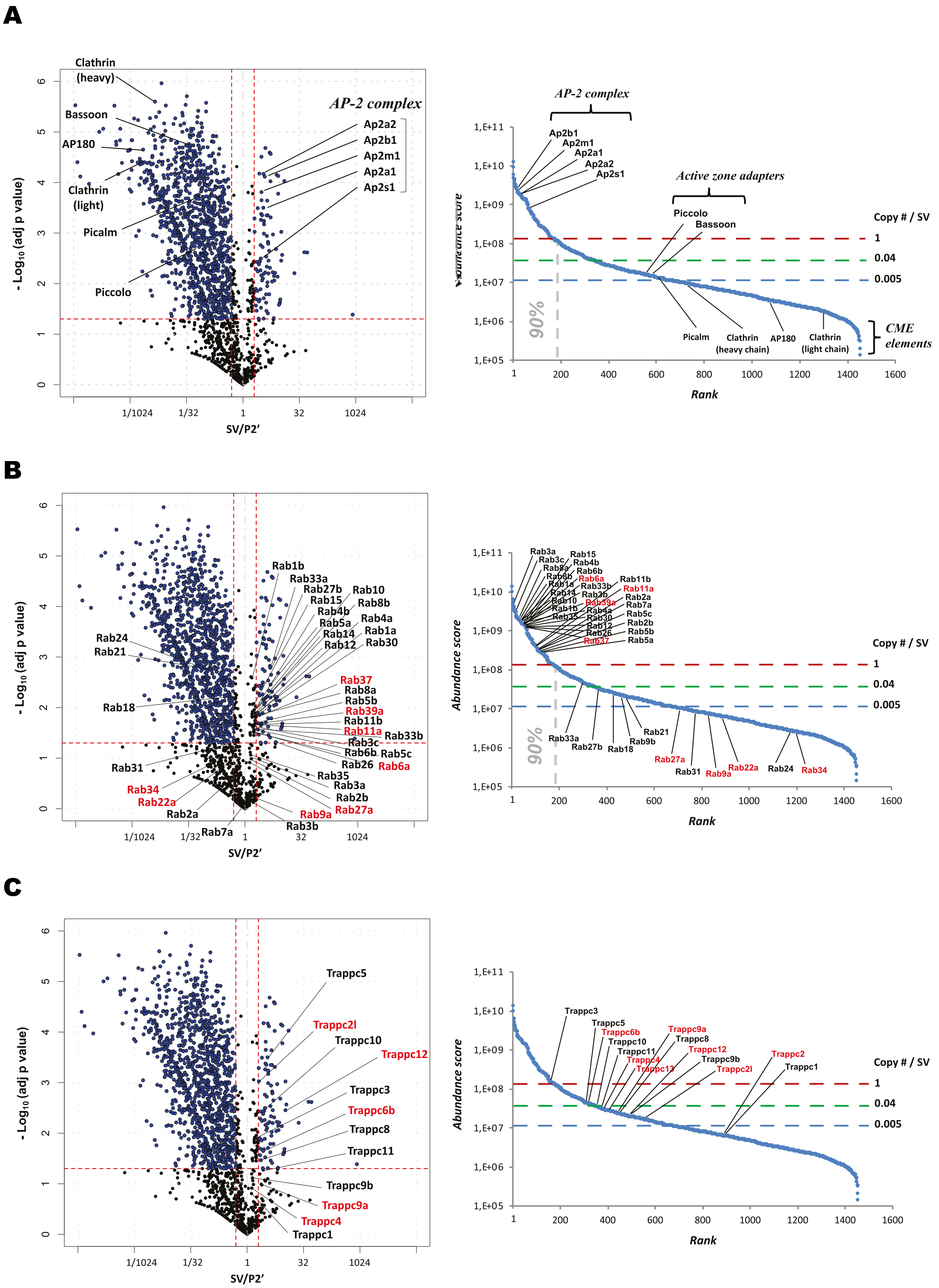
A
Clathrin (heavy)
Bassoon
AP180
Clathrin (light)
Picalm
Piccolo
AP-2 complex
Ap2a2
Ap2b1
Ap2m1
Ap2a1
Ap2s1
- Log10 (adj p value)
SV/P2'
1/1024
1/32
1
32
1024
AP-2 complex
Ap2b1
Ap2m1
Ap2a1
Ap2a2
Ap2s1
Active zone adapters
Piccolo
Bassoon
Copy # / SV
1
0.04
0.005
90%
Picalm
Clathrin (heavy chain)
AP180
Clathrin (light chain)
CME elements
Abundance score
Rank
1,E+11
1,E+10
1,E+09
1,E+08
1,E+07
1,E+06
1,E+05
B
Rab1b
Rab33a
Rab27b
Rab10
Rab15
Rab8b
Rab4b
Rab4a
Rab5a
Rab1a
Rab14
Rab12
Rab30
Rab24
Rab21
Rab18
Rab37
Rab8a
Rab5b
Rab39a
Rab11b
Rab11a
Rab33b
Rab3c
Rab6b
Rab5c
Rab26
Rab6a
Rab35
Rab3a
Rab2b
Rab27a
Rab9a
Rab3b
Rab31
Rab34
Rab22a
Rab2a
Rab7a
Rab3a
Rab3c
Rab15
Rab4b
Rab8a
Rab6b
Rab8b
Rab6a
Rab11b
Rab1a
Rab33b
Rab11a
Rab14
Rab3b
Rab2a
Rab10
Rab39a
Rab7a
Rab1b
Rab4a
Rab5c
Rab35
Rab30
Rab2b
Rab12
Rab5b
Rab26
Rab37
Rab5a
Rab33a
Rab27b
Rab18
Rab21
Rab9b
Rab27a
Rab31
Rab9a
Rab22a
Rab24
Rab34
C
Trappc5
Trappc2l
Trappc10
Trappc12
Trappc3
Trappc6b
Trappc8
Trappc11
Trappc9b
Trappc9a
Trappc4
Trappc1
Trappc3
Trappc5
Trappc6b
Trappc9a
Trappc8
Trappc10
Trappc11
Trappc12
Trappc4
Trappc13
Trappc9b
Trappc2l
Trappc2
Trappc1
1
200
400
600
800
1000
1200
1400
1600

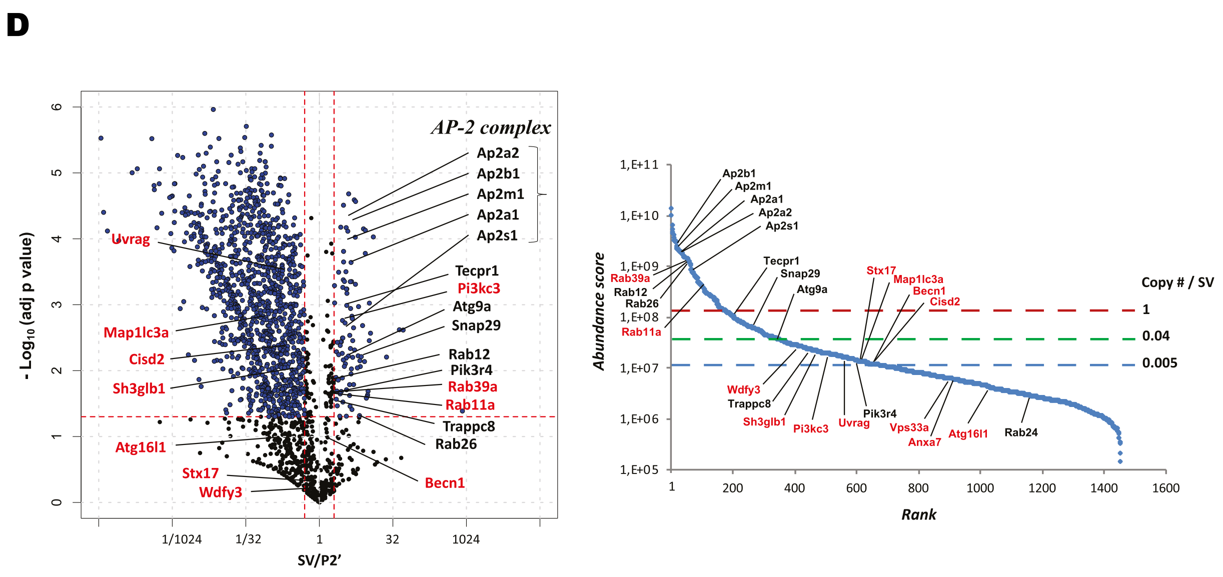

Figure S4. Examples of adapters, trafficking, and autophagy-related proteins detected in the SV proteome with UD proteomics

(A) Position in the SV/P2' volcano plot (*left*) and ranked (iBAQ) abundance plot (*right*) of AP-2 complex, clathrin-mediated endocytosis (CME) elements and other adapter proteins detected in the SV fraction. Active zone (AZ) adapter proteins, such as Piccolo and Bassoon, were detected in low iBAQ abundance ranges, as they are known to interact transiently and to dock with a restricted number of SVs at AZ within the presynaptic compartment. On the other hand, UD proteomics identified all adapter protein complex-2 (AP-2) components as SV-resident and as part of most SVs, whereas CME elements, such as clathrin, AP180 and Picalm, were found in the SV transiently interacting repertoire and in low-abundance ranges. These findings suggest that AP-2 may not undergo uncoating after CME, contrary to assumptions of current models, but it may remain associated with most SVs.

(B) Position in the SV/P2' volcano plot (*left*) and ranked (iBAQ) abundance plot (*right*) of the rab subfamily of small GTPases detected in the SV fraction. A total of 40 rabs were detected in the SV fraction (in red: newly detected by UD proteomics). Many of these were enriched in the SV fraction (7 highly enriched with SV/P2' iBAQ ratio >2, and 18 slightly enriched with iBAQ ratio between 1 and 2) and expressed in most SVs ('canonical repertoire', Takamori *et al.*, 2006), suggesting highly diverse possibilities of trafficking routes for SVs in the presynaptic compartment. Implications of rab diversity in regulation of neurotransmission remain poorly understood.

(C) Position in the SV/P2' volcano plot (*left*) and ranked (iBAQ) abundance plot (*right*) of membrane-tethering Trappc proteins detected in the SV fraction. Most Trappc proteins were SV-resident and widely distributed in the ranked abundance curve. Trappc13 and Trappc2 do not appear in the SV/P2' volcano plot, as they were detected only in the SV fraction.

(D) Position in the SV/P2' volcano plot (*left*) and ranked (iBAQ) abundance plot (*left*) of autophagy-related proteins detected in the SV fraction, showing evidence of a 'vesiculophagy' toolset-comprising adapter, trafficking, tethering, regulatory, and core autophagy complex proteins.

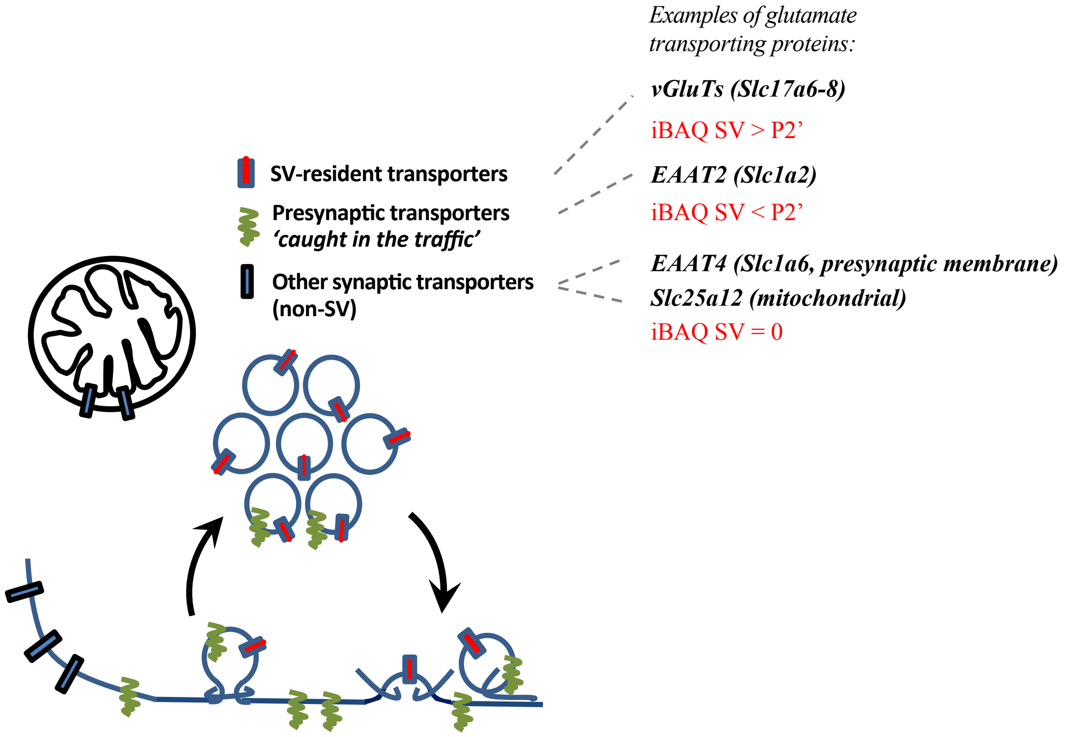

Figure S5. Interpretation of the spatial distribution of transporter proteins detected in the SV and P2' proteomes with UD proteomics
Solute carrier ('slc') transporters are transmembrane proteins that regulate passage of soluble molecules across cellular membranes. In the presynaptic compartment, these proteins were detected either with average iBAQ scores in SV > P2' (SV-resident transporters), with average iBAQ scores in SV < P2' (transiently reside on SV during trafficking), or an iBAQ score in SV = 0 (may never reside on SVs). Transporters having a common substrate may have different localizations and distinct functions within synapses. For example, VGLUT1 (Slc17a7, iBAQ SV > P2') imports glutamate into SVs before neurotransmitter release, whereas EAAT2 (Slc1a2, iBAQ P2' > SV) and EAAT4 (Slc1a6, iBAQ SV = 0) are subtypes of excitatory amino acid transporters involved in the re-uptake of extracellular glutamate from the synaptic cleft into presynapses. Slc25a12 (iBAQ SV = 0) is a transporter protein that shuttles glutamate into mitochondria.

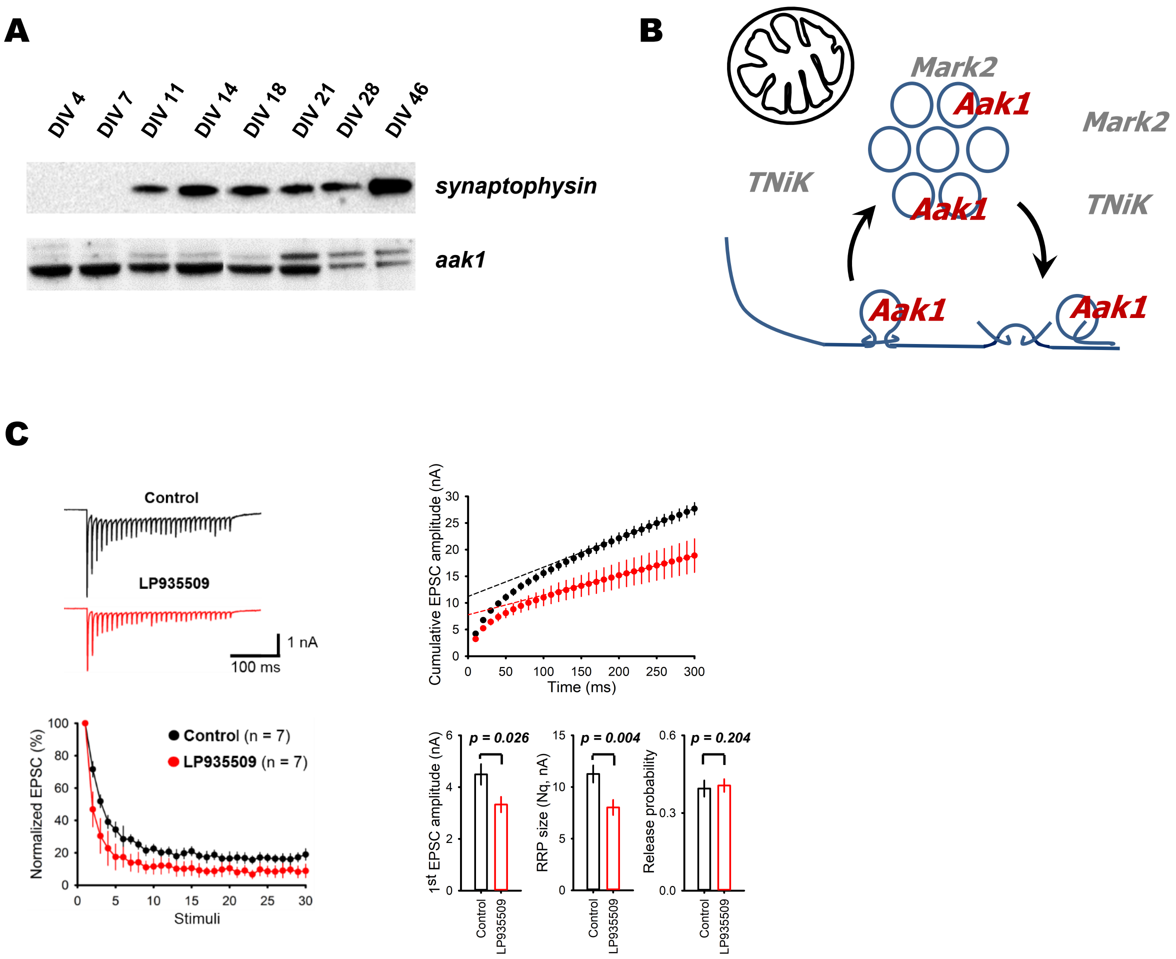

Figure S6. Characterization of SV protein kinase Aak1

(A) Endogenous expression profile of Aak1 and synaptophysin in cultured hippocampal neurons at days *in vitro* (DIV) 4-46. Aak1 was expressed throughout all culture stages whereas synaptophysin, a major SV component, appeared significantly after DIV11.

(B) Interpretation of the spatial distribution of Aak1, Mark2 and TNiK kinases within the presynaptic compartment. Western blot data (Figure S2E), proteomic profiles of SV/P2' ratio (Figure 5A), and confocal images (Figure 5C) suggest that although it is soluble, Aak1 is an SV-resident protein at the presynapse. In contrast, Mark2 may occasionally be found associated with SV (iBAQ scores in P2' > SV), while TNiK, although present in the presynaptic compartment, may not interact with SV (iBAQ scores in SV = 0, see Supplemental database).

(C) Aak1 inhibitor LP935509 (1 μM) loaded directly into presynaptic terminals enhanced STD of EPSCs and reduced estimated RRP size (*Nq*) evoked by a 100-Hz train of stimulation at the calyx of Held synapse.

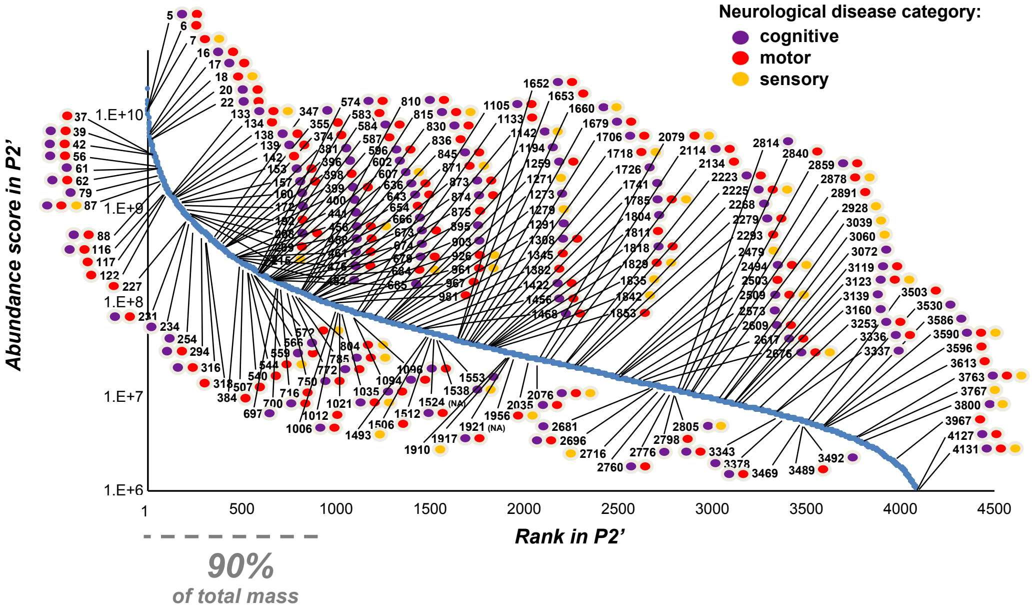

Figure S7. Neurological disease-related proteins detected in SV fraction ranked in the P2' proteome.

Most of the disease-related SV proteins (55%) are found in the low abundance ranges of the synaptosomal proteome (i.e. beyond abundance rank 875; rank 1 to 875 = 90% of the total P2' mass).

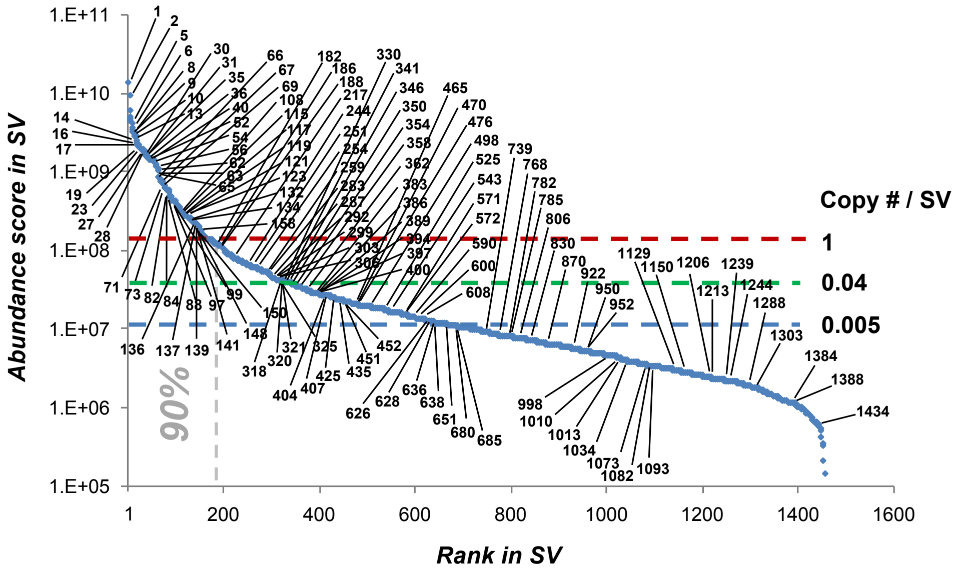

Figure S8. 'SV-residents' ranked in the SV proteome.

Position of all the 134 proteins detected as SV-residents in the ranked abundance curve of the UD-SV proteome. Proteins that were found with significant >2 fold enrichment in SV over P2' fractions are displayed (see Supplemental database). Of 134 SV-resident proteins, 86 are of low abundance (<1 average copy number per SV). These proteins include vesicular transporters for monoamines and acetylcholine neurotransmitters, present in only a small percentage of brain synapses.

Protein	MW (kDa)	native/heavy	Absolute Quantity (ug)	Average #/SV
All	*NA*	*NA*	50	*NA*
Synaptotagmin 1	47	14.88	1.5	15 *
Rab3A	25	5.61	0.56	10.5
Aak1	104	3.35	0.34	1.53
Mover	30	0.57	0.06	0.94
Atg9A	94	0.08	0.008	0.04
Git1	85	0.01	0.001	0.006

(** from Takamori et al 2006*)

Table S1. Absolute quantity and copy number per SV for selected proteins.
Absolute quantification was performed by mixing known quantities of heavy-isotope synthetic peptides (0.1 µg) with 50 µg of digested SV proteins. The exact quantity of the peptide of interest (native) was calculated using the intensity of the heavy-isotope-labeled standard peptide (heavy) (Figure S3): Absolute quantity (µg) = 0.1 x (native/heavy peak areas)
Average copy number of a protein per SV (#/SV) was estimated using synaptotagmin 1 (Syt1), a transmembrane SV-resident protein of molecular weight (MW) = 47 kDa, as a reference (15 Syt1 per SV, estimated by Takamori *et al.,* 2006):
Protein #/SV = absolute quantity/MW x [(Syt1 #/SV) x (Syt1 MW) / (Syt1 absolute quantity)].

	transporter protein name	substrate	rank in SV proteome
known	vGluT1	glutamate	8
	vGluT2	glutamate	36
	vGAT	GABA, glycine	40
	VAT-1	monoamines	123
	vMAT2	monoamines	303
	Slc5a7 (High affinity choline transporter 1)	choline	383
	vAchT	acetylcholine	386
	SVOP	?	486
new	Nipal3 (NIPA-like protein 3)	magnesium	182
	Slc35g2 (Solute carrier family 35 member G2)	?	244
	Protein Mfsd6	?	325
	Slc22a17 (Solute carrier family 22 member 17)	iron ?	450
	Lmbrd2 (LMBR1 domain-containing protein 2)	vitamin B12 ?	452
	Slc36a3 (Proton-coupled amino acid transporter 3)	glycine	470
	Slc7a4 (Cationic amino acid transporter 4)	arginine, lysine, ornithine	641
	Slc10a4 (Sodium/bile acid cotransporter 4)	bile acids ?	830
	Slc45a1 (Proton-associated sugar transporter A	glucose (pH gradient)	1388

Table S2. Transporters and their putative substrates newly detected as SV-residents using UD proteomics.

Animal species (common names)	RefSeq	Protein submitted name	Uniprot ID	gene name
Rattus norvegicus (Norway rat)	NP_001020140.2	uncharacterized protein C7orf43 homolog	A0A0G2KAX2	RGD1305455
Mus musculus (house mouse)	NP_694801.2	uncharacterized protein C7orf43 homolog	Q3UTZ3	cDNA sequence BC037034
Delphinapterus leucas (beluga whale)	XP_022443585.1	uncharacterized protein C7orf43 homolog isoform X1	*NA*	chromosome unknown C7orf43 homolog (CUNH7orf43)
Loxodonta africana (African savanna elephant)	XP_003422547.2	Chromosome 7 open reading frame 4	*NA*	chromosome unknown C7orf43 homolog (CUNH7orf43)
Callithrix jacchus (white-tufted-ear marmoset)	XP_008980987.1	Uncharacterized protein C7orf43	U3D2Q3	chromosome 2 C7orf43 homolog (C2H7orf43)
Pongo abelii (Sumatran orangutan)	XP_009240892.1	Chromosome 7 open reading frame 43	H2PLM0	chromosome 7 C7orf43 homolog (C7H7orf43)
Homo sapiens (human)	NP_060745.3	uncharacterized protein C7orf43	Q8WVR3	chromosome 7 open reading frame 43 (C7orf43)
Pelodiscus sinensis (Chinese soft-shelled turtle)	XP_014431082.1	uncharacterized protein C7orf43 homolog	*NA*	chromosome unknown open reading frame, human C7orf43
Pseudopodoces humilis (Tibetan ground-tit bird)	XP_014117365.1	uncharacterized protein C7orf43 homolog isoform X1	*NA*	chromosome unknown open reading frame, human C7orf43
Amphiprion ocellaris (Clown anemonefish)	XP_023119364.1	uncharacterized protein C7orf43 homolog isoform X2	*NA*	C7orf43 homolog
Branchiostoma floridae (Florida lancelet)	XP_002592972.1	hypothetical protein BRAFLDRAFT_65558	C3ZE27	BRAFLDRAFT_65558

Table S3. Reference sequences and accession numbers used for the amino acid sequence comparison of RGD1305455 homologs.

#	b	b-H2O	b-NH3	b (2+)	Seq	y	y-H2O	y-NH3	y (2+)	#
1	100.08	82.07	83.05	50.54	V					8
2	213.16	195.15	196.13	107.08	L	791.51	773.50	774.48	396.26	7
3	312.23	294.22	295.20	156.61	V	678.43	660.42	661.40	339.71	6
4	411.30	393.29	394.27	206.15	V	579.36	561.35	562.33	290.18	5
5	540.34	522.33	523.31	270.67	E	480.29	462.28	463.26	240.65	4
6	637.39	619.38	620.37	319.20	P	351.25	333.24	334.22	176.12	3
7	736.46	718.45	719.43	368.73	V	254.19	236.18	237.17	127.60	2
8					K(+8.01)	155.13	137.12	138.10	78.06	1

#	b	b-H2O	b-NH3	b (2+)	Seq	y	y-H2O	y-NH3	y (2+)	#
1	99.85	82.07	83.05	50.54	V					8
2	213.16	195.15	196.13	107.08	L	783.49	765.49	766.47	392.25	7
3	312.23	294.22	295.20	156.61	V	670.41	652.40	653.39	335.71	6
4	411.21	393.29	394.27	206.15	V	571.34	553.33	554.32	286.17	5
5	540.34	522.33	523.31	270.67	E	472.27	454.27	455.27	236.64	4
6	637.39	619.38	620.37	319.20	P	343.23	325.22	326.17	172.15	3
7	736.46	718.45	719.43	368.73	V	246.18	228.17	229.15	123.59	2
8					K	147.11	129.10	130.09	74.06	1

Table S4. Tables of fragment ion masses expected from heavy and native VLVVEPVK peptides.

Masses were generated *in silico* with PEAK (v7, Bioinformatics Solutions Inc.). Colored masses indicate those from matched ion MS2 spectra observed in our experiments (Figure 4C). Note that detected y-ions masses (in red) that include the C-terminal amino acid are 8 Daltons heavier in the heavy peptide (upper table) compared to the native peptide (lower table).

Protein name	Uniprot ID	gene name	heavy peptide
Uncharacterized Protein RGD1305455	A0A0G2KAX2	RGD1305455	VLVVEPVK
			EVEAPEVR
			LVWTPEHAQAGK
			RNHLYLGETVR
Protein Mfsd6	D3ZCJ3	mfsd6	GKEMEIPQVER
Sodium/bile acid cotransporter 4	F1LQG5	slc10a4	EALDEDDDTDISYKK
Proton-associated sugar transporter A	Q566E3	Slc45a1	YGSFISR
High affinity choline transporter 1	G3V7G1	Slc5a7	QSLTLSSTFTNK
Solute carrier family 35 member G2	Q5M7A3	Slc35g2	TLFGTMDTQPPR
			GQFQSFAEK
NIPA-like protein 3	D3ZZI0	Nipal3	STPGVPYR
			GMTVQPDLK
LMBR1 domain-containing protein 2	D3ZUP8	Lmbrd2	SGGFSITGK
Solute carrier family 22 member 17	Q9P290	Slc22a17	LLPEVLR
			QIEEAQSVLR
Cationic amino acid transporter 4	B5DFJ0	Slc7a4	LKPLEESSTETSLRR

Table S5. Heavy peptides used to confirm the presence of newly identified SV-resident proteins.

Peptide tracking experiments using these synthesized heavy peptides, as described in Figure 4C gave uniformly positive results, providing additional evidence of a hitherto undetected SV proteome revealed by UD proteomics.

References:

1. B. MacLean *et al.*, Skyline: an open source document editor for creating and analyzing targeted proteomics experiments. *Bioinformatics* **26**, 966-968 (2010).

2. F. Koopmans *et al.*, SynGO: an evidence-based, expert-curated knowledge base for the synapse. *Neuron* **103**, 217-234 (2019).

3. H. Hioki *et al.*, High-level transgene expression in neurons by lentivirus with Tet-Off system. *Neurosci Res* **63**, 149-154 (2009).

4. Y. Egashira, M. Takase, S. Takamori, Monitoring of vacuolar-type H+ ATPase-mediated proton influx into synaptic vesicles. *J Neurosci* **35**, 3701-3710 (2015).

5. J. H. Kim *et al.*, High cleavage efficiency of a 2A peptide derived from porcine teschovirus-1 in human cell lines, zebrafish and mice. *PLoS One* **6**, e18556 (2011).

6. C. Chen, H. Okayama, High-efficiency transformation of mammalian cells by plasmid DNA. *Mol Cell Biol* **7**, 2745-2752 (1987).

7. G. M. Beaudoin, 3rd *et al.*, Culturing pyramidal neurons from the early postnatal mouse hippocampus and cortex. *Nat Protoc* **7**, 1741-1754 (2012).

8. M. Wiznerowicz, D. Trono, Conditional suppression of cellular genes: lentivirus vector-mediated drug-inducible RNA interference. *J Virol* **77**, 8957-8961 (2003).

9. R. H. Kutner, X. Y. Zhang, J. Reiser, Production, concentration and titration of pseudotyped HIV-1-based lentiviral vectors. *Nat Protoc* **4**, 495-505 (2009).

10. S. K. Ultanir *et al.*, Chemical genetic identification of NDR1/2 kinase substrates AAK1 and Rabin8 Uncovers their roles in dendrite arborization and spine development. *Neuron* **73**, 1127-1142 (2012).

11. S. Okuda *et al.*, jPOSTrepo : an international standard data repository for proteomes. *Nucleic Acids Research* **45** (D1), D1107-D1111 (2017).
